# Evaluation of the Xpert MTB Host Response assay for the triage of patients with presumed pulmonary tuberculosis: a prospective diagnostic accuracy study in Viet Nam, India, the Philippines, Uganda, and South Africa

**DOI:** 10.1016/S2214-109X(23)00541-7

**Published:** 2024-02

**Authors:** Ankur Gupta-Wright, Huy Ha, Shima Abdulgadar, Rebecca Crowder, Jerusha Emmanuel, Job Mukwatamundu, Danaida Marcelo, Patrick P J Phillips, Devasahayam Jesudas Christopher, Nguyen Viet Nhung, Grant Theron, Charles Yu, Payam Nahid, Adithya Cattamanchi, William Worodria, Claudia M Denkinger, on behalf of the R2D2 TB Network

**Affiliations:** Division of Infectious Disease and Tropical Medicine and German Centre for Infection Research, Heidelberg University Hospital, Heidelberg, Germany; Institute for Global Health, University College London, London, UK; Hanoi Lung Hospital, Hanoi, Viet Nam; DSI-NRF Centre of Excellence for Biomedical Tuberculosis Research, South African Medical Research Council Centre for Tuberculosis Research, Division of Molecular Biology and Human Genetics, Faculty of Medicine and Health Sciences, Stellenbosch University, Cape Town, South Africa; UCSF Center for Tuberculosis, San Francisco General Hospital, University of California San Francisco, San Francisco, CA, USA; Department of Pulmonary Medicine, Christian Medical College, Vellore, India; World Alliance for Lung and Intensive Care Medicine in Uganda, Kampala, Uganda; De La Salle Medical Health Sciences Institute, Dasmariñas City, Cavite, Philippines; UCSF Center for Tuberculosis, San Francisco General Hospital, University of California San Francisco, San Francisco, CA, USA; Division of Pulmonary and Critical Care Medicine, University of California San Francisco, San Francisco, CA, USA; Department of Pulmonary Medicine, Christian Medical College, Vellore, India; Vietnam National Lung Hospital, NTP, HMU, Hanoi, Viet Nam; DSI-NRF Centre of Excellence for Biomedical Tuberculosis Research, South African Medical Research Council Centre for Tuberculosis Research, Division of Molecular Biology and Human Genetics, Faculty of Medicine and Health Sciences, Stellenbosch University, Cape Town, South Africa; De La Salle Medical Health Sciences Institute, Dasmariñas City, Cavite, Philippines; UCSF Center for Tuberculosis, San Francisco General Hospital, University of California San Francisco, San Francisco, CA, USA; Division of Pulmonary and Critical Care Medicine, University of California San Francisco, San Francisco, CA, USA; UCSF Center for Tuberculosis, San Francisco General Hospital, University of California San Francisco, San Francisco, CA, USA; Division of Pulmonary Diseases and Critical Care Medicine, University of California Irvine, Irvine, CA, USA; World Alliance for Lung and Intensive Care Medicine in Uganda, Kampala, Uganda; Division of Pulmonology, Mulago National Referral Hospital, Kampala, Uganda; Division of Infectious Disease and Tropical Medicine and German Centre for Infection Research, Heidelberg University Hospital, Heidelberg, Germany

## Abstract

**Background:**

Non-sputum-based triage tests for tuberculosis are a priority for ending tuberculosis. We aimed to evaluate the diagnostic accuracy of the late-prototype Xpert MTB Host Response (Xpert HR) blood-based assay.

**Methods:**

We conducted a prospective diagnostic accuracy study among outpatients with presumed tuberculosis in outpatient clinics in Viet Nam, India, the Philippines, Uganda, and South Africa. Eligible participants were aged 18 years or older and reported cough lasting at least 2 weeks. We excluded those receiving tuberculosis treatment in the preceding 12 months and those who were unwilling to consent. Xpert HR was performed on capillary or venous blood. Reference standard testing included sputum Xpert MTB/RIF Ultra and mycobacterial culture. We performed receiver operating characteristic (ROC) analysis to identify the optimal cutoff value for the Xpert HR to achieve the target sensitivity of 90% or more while maximising specificity, then calculated diagnostic accuracy using this cutoff value. This study was prospectively registered with ClinicalTrials.gov, NCT04923958.

**Findings:**

Between July 13, 2021, and Aug 15, 2022, 2046 adults with at least 2 weeks of cough were identified, of whom 1499 adults (686 [45·8%] females and 813 [54·2%] males) had valid Xpert HR and reference standard results. 329 (21·9%) had microbiologically confirmed tuberculosis. Xpert HR had an area under the ROC curve of 0·89 (95% CI 0·86–0·91). The optimal cutoff value was less than or equal to −1·25, giving a sensitivity of 90·3% (95% CI 86·5–93·3; 297 of 329) and a specificity of 62·6% (95% CI 59·7–65·3; 732 of 1170). Sensitivity was similar across countries, by sex, and by subgroups, although specificity was lower in people living with HIV (45·1%, 95% CI 37·8–52·6) than in those not living with HIV (65·9%, 62·8–68·8; difference of 20·8%, 95% CI 13·0–28·6; p<0·0001). Xpert HR had high negative predictive value (95·8%, 95% CI 94·1–97·1), but positive predictive value was only 40·1% (95% CI 36·8–44·1). Using the Xpert HR as a triage test would have reduced confirmatory sputum testing by 57·3% (95% CI 54·2–60·4).

**Interpretation:**

Xpert HR did not meet WHO minimum specificity targets for a non-sputum-based triage test for pulmonary tuberculosis. Despite promise as a rule-out test that could reduce confirmatory sputum testing, further cost-effectiveness modelling and data on acceptability and usability are needed to inform policy recommendations.

**Funding:**

National Institute of Allergy and Infectious Diseases of the US National Institutes of Health.

## Introduction

Tuberculosis was the second leading cause of infectious disease mortality after COVID-19 in 2022, and a lack of diagnosis is responsible for the largest gap in the tuberculosis care cascade.^1^ Approximately 30% of the estimated 10·6 million people with tuberculosis in 2022 were undiagnosed. Rapid scale-up of improved tuberculosis diagnostic tools is a priority to achieve the End TB Strategy targets by 2035.^2^ To guide development, WHO issued a series of target product profiles in 2014,^3^ with one of the highest priority targets being a non-sputum-based triage test to rule out tuberculosis disease (ie, having a high negative predictive value). A triage test with a minimum sensitivity of 90% and specificity of 70% that also meets the predefined operational targets could potentially help reduce underdiagnosis and misdiagnosis of tuberculosis, reduce delays in starting tuberculosis treatment, and lower the cost and infrastructure requirements of current gold-standard, sputum-based tests for tuberculosis in high-burden settings.^4^ No triage test currently meets these targets.

Several technologies offer promise, including host blood transcriptomic signatures.^5^ Sweeney and colleagues^6^ validated a three-gene host blood signature using blood mRNA based on expression of markers that form part of the host inflammatory response and can discriminate tuberculosis disease from other diseases. The signature has been developed into an automated, blood-based quantitative PCR test using the GeneXpert platform (Cepheid, Sunnyvale, CA, USA). The test gives a TB Score based on the cycle threshold of expressed genes. Early studies from stored samples^7,8^ and interim results from a multisite study^9^ suggest that diagnostic accuracy could reach the WHO target product profile minimum accuracy threshold for a tuberculosis triage test.

Large, prospective studies are needed to validate performance of the Xpert HR as a triage test, including among key subpopulations, and inform WHO guidelines and policy makers. Therefore, we aimed to prospectively evaluate the diagnostic accuracy of the late-prototype Xpert MTB Host Response (Xpert HR; Cepheid, Sunnyvale, CA, USA) assay, a first-in-class host RNA transcriptomic signature-based test, against a microbiological reference standard as a triage test for pulmonary tuberculosis and compared its performance with the WHO target product profiles.

## Methods

### Study design and participants

We conducted a prospective diagnostic accuracy study among outpatients with presumed tuberculosis in outpatient clinics in five countries (Viet Nam, India, the Philippines, Uganda, and South Africa) as part of the Rapid Research in Diagnostics Development (R2D2) TB Network (appendix 3 p 5). We screened consecutive adults (≥18 years old) presenting to clinics for any reason and included those with cough lasting at least 2 weeks. We excluded those treated for tuberculosis infection or disease in the past 12 months, having taken antimycobacterial antibiotics in the past 2 weeks, or unable or unwilling to return for follow-up or provide informed consent.

All study-related activities were approved by the University of California San Francisco Institutional Review Board (20–32670), the University of Heidelberg Ethics Committee of the Medical Faculty (S-539/2020), and research ethics committees in each country (appendix 3 p 5). All participants provided written informed consent. This study conformed to the Standards for Reporting of Diagnostic Accuracy Studies reporting guidelines (appendix 3 p 23).

### Procedures

Eligible and consenting participants had detailed demographic, tuberculosis symptom, and medical history recorded using a standardised case report form. Sex data were collected by self-reporting. All participants had fingerprick or venous blood collected for HIV and diabetes screening (using glycated haemoglobin [HbA_1c_]), and up to three spot sputum samples collected for reference standard testing (sputum was induced if unable to expectorate spontaneously). Reference standard testing was with Xpert MTB/RIF Ultra (Xpert Ultra; Cepheid, Sunnyvale, CA, USA), with repeat testing if the initial result was trace positive, invalid, or indeterminate, and two cultures using liquid mycobacterium growth indicator tube (MGIT, Becton Dickinson, Franklin Lakes, NJ, USA) media (culture only performed if sputum Xpert Ultra was not positive). Solid media culture was only performed for participants when MGIT tubes were not available. Fingerprick (South Africa only) or venous (all sites) blood samples were also collected for Xpert HR testing (appendix 3 pp 6–7). Study data were collected and managed using REDCap electronic data capture tools, hosted at the University of California San Francisco.^10,11^

For the Xpert HR, 100 μL of venous or capillary blood was collected and transferred to the cartridge and the assay run on the GeneXpert platform as per the manufacturer’s instructions. Results were available within 50 min. In the case of an invalid result, the test was repeated if sufficient sample was available. The test uses reverse transcriptase PCR (RT-PCR) to evaluate the mRNA levels of four target genes, including *GBP5, DUSP3, KLF2,* and *TBP*. Cycle threshold values for these individual four genes, as well as the TB Score calculated using the formula (cycle threshold of *GBP5* + cycle threshold of *DUSP3*)/2 – cycle threshold of *TBP* were recorded. The calculated TB Score differs from the one used in previous studies, which included *KLF2* instead of *TBP* in the three-gene signature. This change was recommended by the manufacturer due to improved stability and performance across blood collection methods. No prespecified cutoff value was provided by the manufacturer.

### Outcomes

The primary outcome was diagnostic accuracy of Xpert HR compared with a microbiological reference standard defined by any positive (very low grade or higher) sputum Xpert Ultra result, two positive (trace or higher) sputum Xpert Ultra results, or any positive culture result in liquid (MGIT) or solid media (appendix 3 p 8).^12^ Participants with no positive Xpert Ultra result and two negative culture results were considered negative by microbiological reference standard. The initial protocol in India included only one culture from Aug 16, to Nov 18, 2021. Therefore, participants enrolled in India during this period were considered tuberculosis negative by microbiological reference standard if their sputum Xpert Ultra result and their single sputum culture result were negative.

Secondary outcomes were net benefit of Xpert HR as a triage test compared with testing all participants with sputum-based tests, diagnostic accuracy compared with a simplified sputum Xpert Ultra reference standard, proportion of invalid results, positive and negative predictive values, and time to testing from sample collection. For the sputum Xpert Ultra reference standard, tuberculosis positive was defined as having any positive (very low or higher grade) or two positive (trace or higher grade) sputum Xpert Ultra results, and tuberculosis negative as having a negative sputum Xpert Ultra result. For both reference standards, clinical information and Xpert HR results were not available to those assessing the reference standard. Post-hoc analysis compared original TB Score accuracy using *KLF2* with the updated TB Score using *TBP*.

### Statistical analysis

We performed receiver operating characteristic (ROC) analysis of the Xpert HR TB Score against the microbiological reference standard to identify the optimal cutoff value to achieve the target product profile sensitivity of 90% or more for a triage test. Using this cutoff value, we then calculated specificity, and positive and negative predictive values with respect to the reference standards with binomial 95% CIs. Subgroup analyses were conducted by country, blood collection method, HIV status, and diabetes status and were prespecified in the statistical analysis plan. ROC curves were generated to visualise diagnostic accuracy across all possible cutpoints and to show differences by subgroup. Area under the ROCs (AUCs) were compared for subgroups using DeLong’s test, and binomial CIs calculated (using the roctab command in Stata). The total planned sample size of 1500 participants (approximately 300 per country) was chosen to give a lower 95% CI exceeding 80% sensitivity within country, and exceeding 86% across countries, assuming a true sensitivity of 90% and tuberculosis prevalence of 20%. Positive predictive values and negative predictive values were calculated based on sensitivity, specificity, and prevalence in the cohort, and modelled for a range of tuberculosis prevalences (5–30%) using Bayes’ Theorem.^13^ Proportions were compared using χ^2^ tests. All analyses were conducted using Stata version 17.1 or R version 4.3.0.

Decision curve analysis (to assess the potential clinical utility of Xpert HR) yields the net benefit of a decision based on the true positives or negatives (benefit), offset by the false positive or negative rate (harm), and is weighted by the risk–benefit ratio of the diagnostic (threshold probability).^14,15^ The threshold probability describes how clinicians and people with presumptive tuberculosis value different outcomes and can be defined as the minimum probability of tuberculosis disease at which further testing should be performed. For example, patients or clinicians might be very concerned about missing tuberculosis and therefore want to do further tuberculosis testing (lower threshold probability [eg, 5%], which is equivalent to a risk–benefit ratio of 1:19, or the risk of missing a tuberculosis diagnosis is 20 times greater than unnecessary investigations for tuberculosis). By contrast, clinicians might be less worried about tuberculosis and not want to do further testing (higher threshold probability [eg, 20%], a risk–benefit ratio of 1:4, or the risk of missing tuberculosis is 5 times greater than unnecessary investigations for tuberculosis). We considered 30% to be the clinically relevant upper limit of threshold probability because any greater threshold probability and further testing would be likely to be done by everyone. Given the default strategy is to test all individuals who meet specific symptom criteria for tuberculosis, this analysis expresses net benefit as unnecessary (sputum-based) tuberculosis testing avoided. Analyses used the dca command in Stata version 17.1. This study was prospectively registered with ClinicalTrials.gov, NCT04923958.

### Role of the funding source

The funder of the study had no role in study design, data collection, data analysis, data interpretation, or writing of the report.

## Results

**Figure 1:**
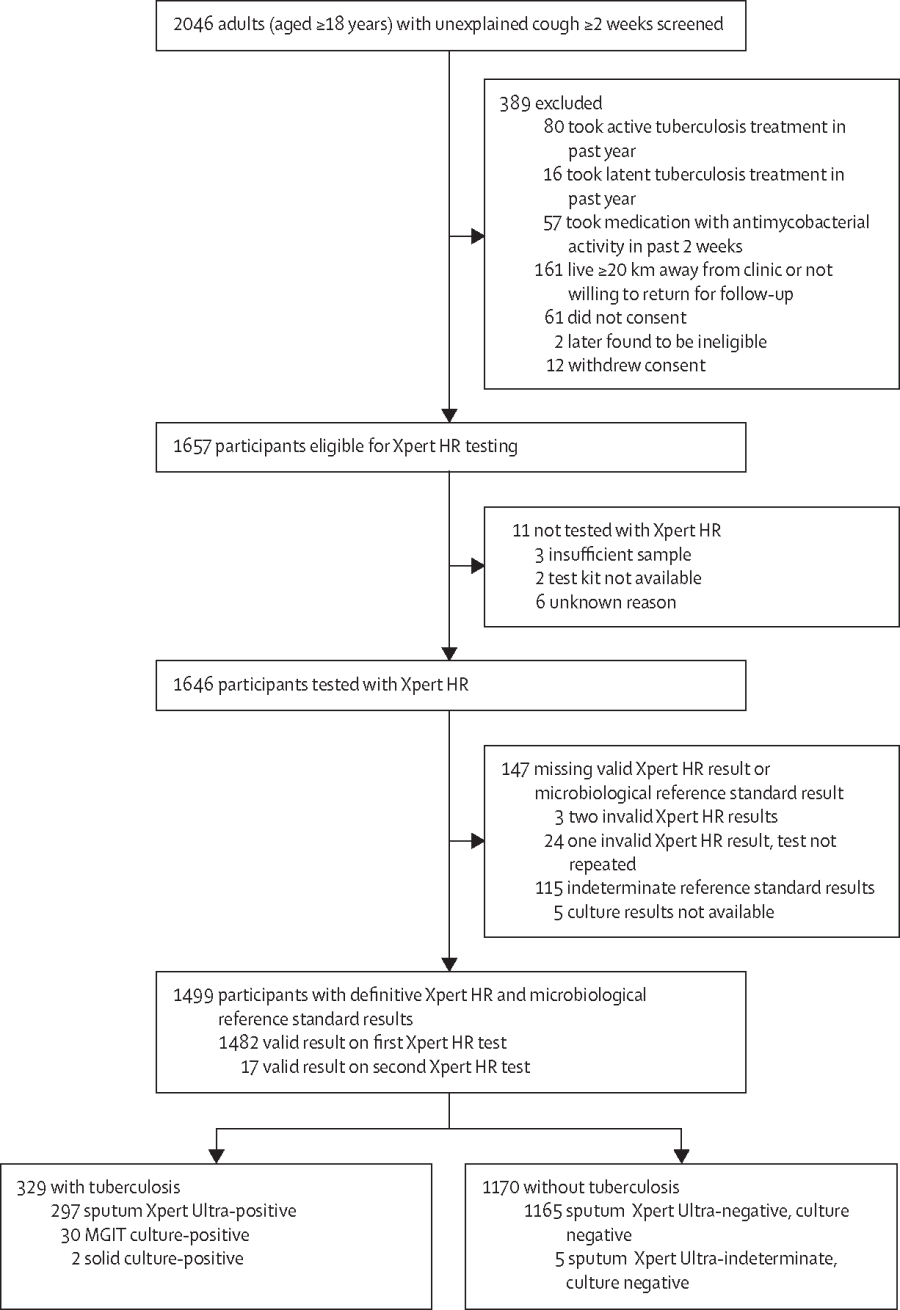
Participant flow chart Reasons for indeterminate reference standard results are detailed in appendix 3 (p 10). MGIT=mycobacterium growth indicator tube. Xpert HR=Xpert MTB Host Response.

Between July 13, 2021, and Aug 15, 2022, we identified 2046 adults with at least 2 weeks of cough. 389 (19·0%) were excluded, and of 1657 eligible participants, 11 (0·7%) were not tested with Xpert HR (figure 1). 27 (1·6%) of 1646 participants had an invalid Xpert HR result and 120 (7·3%) had indeterminate microbiological reference standard results (figure 1). Of the 1499 assessable participants included in the primary analysis, 686 (45·8%) were female, 813 (54·2%) were male, the median age was 41·0 years (IQR 29·0–54·0), 229 (15·3%) were living with HIV (median CD4 count 399 cells per μL [IQR 206–667]), and 202 (13·5%) had diabetes (HbA_1c_ ≥6·5%; table). Baseline characteristics differed across countries, including sex and proportions living with HIV and diabetes. Race and ethnicity data were not collected.

Of the 1499 participants analysed, 329 (21·9%) had confirmed tuberculosis according to the microbiological reference standard (figure 1, table). Proportions with confirmed tuberculosis varied by country, ranging from 29 (8·9%) of 325 in the Philippines to 108 (38·0%) of 284 in Viet Nam, and was greater in participants with diabetes than those without (60 [29·7%] of 202 *vs* 269 [20·7%] of 1297; p=0·0040) but did not differ between those living with HIV and those not living with HIV (45 [19·7%] of 229 *vs* 283 [22·3%] of 1267; p=0·37; appendix 3 p 11). 297 (19·8%) of 1499 participants were sputum Xpert Ultra positive.

**Figure 2:**
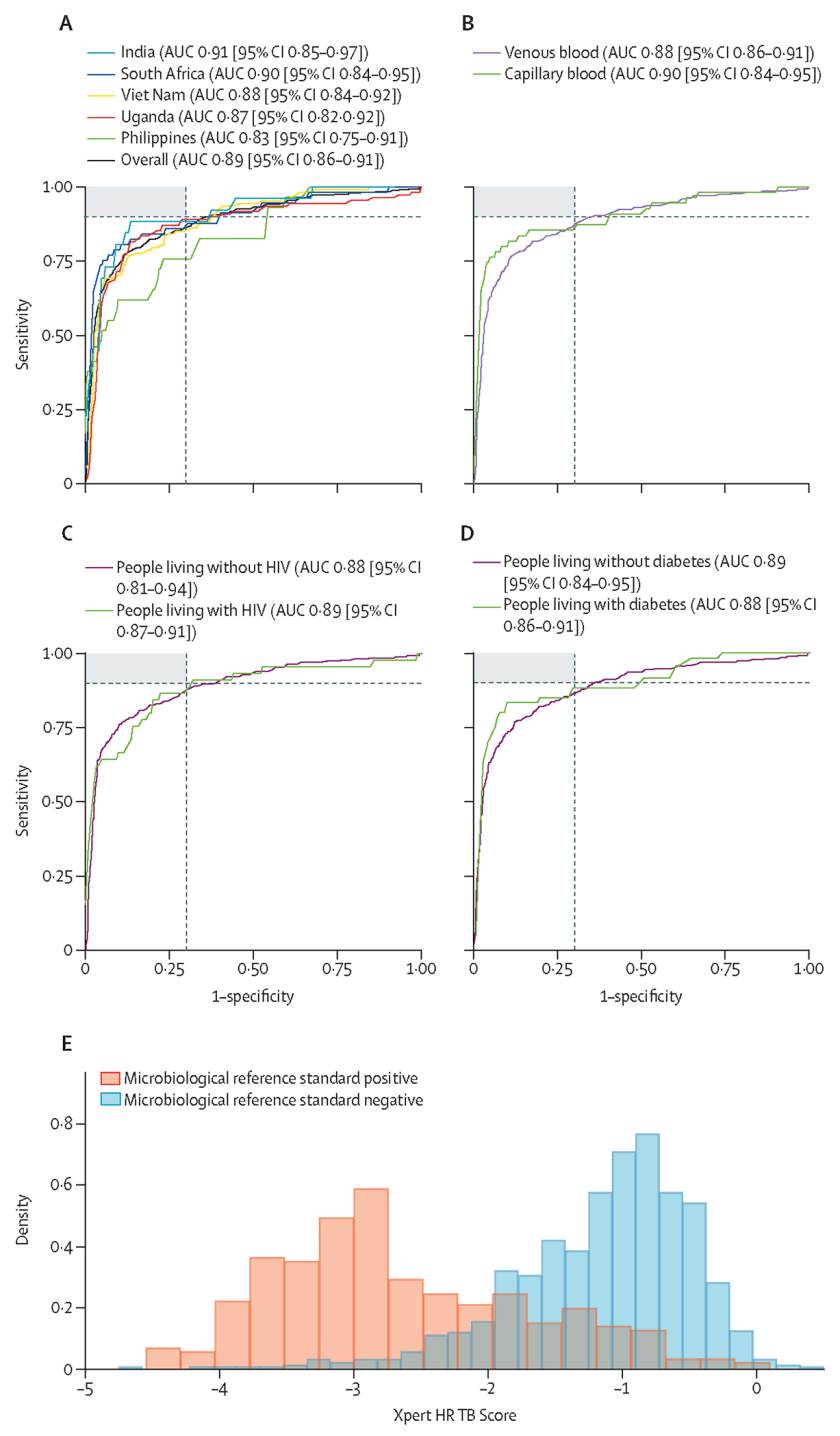
Xpert HR TB Score ROC curve analysis against microbiological reference standard (A) The overall ROC curve for TB Score and by country. (B) ROC curves by blood collection method. (C) ROC curves by HIV status. (D) ROC curves by diabetes status. The shaded region in charts A–D shows cutoff values that meet both the WHO target product profile minimal target sensitivity (≥90%) or specificity (≥70%), the dashed grey lines show the WHO target product profile minimal targets. AUCs and 95% CIs overall, and by country and subgroup, are in appendix 3 (p 14). (E) Xpert HR TB Score by microbiological reference standard. Median TB Score was −2·85 (IQR −3·28 to −2·18) for participants positive by microbiological reference standard, and −1·05 (IQR −1·50 to −0·70) for participants negative by microbiological reference standard. AUC=area under the ROC. ROC=receiver operating characteristic. Xpert HR=Xpert MTB Host Response.

286 (19·1%) of 1499 participants with valid Xpert HR results were tested using capillary blood, with the rest tested using venous blood. TB Score and cycle threshold values for each gene marker for participants with and without tuberculosis are shown in figure 2 and appendix 3 p 12. Lower TB Score was associated with markers of increased tuberculosis severity including higher semiquantitative bacterial load on Xpert Ultra, lower BMI, and higher C-reactive protein (appendix 3 p 14).

**Figure 3:**
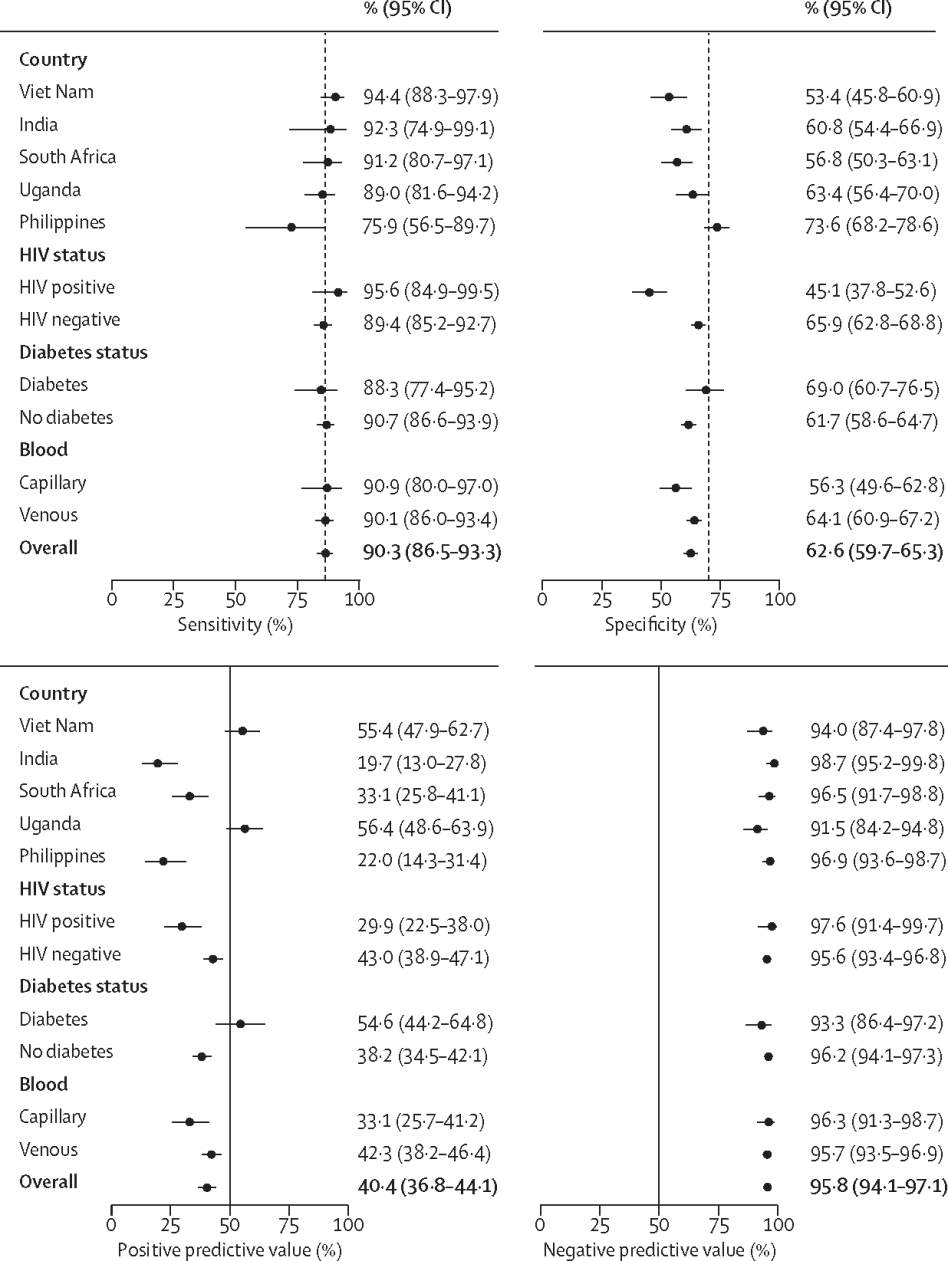
Diagnostic accuracy of the Xpert HR compared with the microbiological reference standard Sensitivity, specificity, positive predictive value, and negative predictive value of the Xpert HR are compared with the microbiological reference standard overall and by subgroup. Vertical dashed line shows WHO target product profile minimum values for sensitivity (≥90%) and specificity (≥70%). Xpert HR=Xpert MTB Host Response.

When evaluated against the microbiological reference standard, Xpert HR had an AUC of 0·89 (95% CI 0·86–0·91). AUCs were similar across countries and by sex, diabetes, and HIV status (figure 2, appendix 3 p 14). The optimal cutoff value for the TB Score that achieved a sensitivity of 90% or more and maximised specificity across the entire dataset was less than or equal to −1·25. At this cutoff value, sensitivity was 90·3% (95% CI 86·5–93·3; 297 of 329) and specificity was 62·6% (59·7–65·3; 732 of 1170; appendix 3 p 18). Sensitivity was similar between countries (p=0·075), except for the Philippines, where sensitivity was lower (75·9%, 95% CI 56·5–89·7; 22 of 29; p<0·0001), but specificity was higher (73·6%, 68·2–78·6; 218 of 296; p<0·0001; figure 3).

Sensitivity was also similar by sex and across other subgroups, but specificity was lower in people living with HIV (45·1%, 95% CI 37·8–52·6) than in those living without HIV (65·9%, 62·8–68·8; difference of 20·8%, 95% CI 13·0–28·6; p<0·0001; appendix 3 p 18). Specificity was also lower with capillary blood sampling (56·3%, 95% CI 49·6–62·8) than with venous blood sampling (64·1%, 60·9–67·2; difference of 7·8 percentage points, 95% CI 1·3–14·3; p=0·015), although capillary blood sampling and venous sampling had similar specificities in people not living with HIV (64·4%, 95% CI 55·8–72·5 *vs* 66·1%, 62·8–69·3; p=0·67; appendix 3 p 15).

Overall, using a TB Score cutoff value of less than or equal to −1·25, Xpert HR had high negative predictive value (95·8%, 95% CI 94·1–97·1) in the study population, and negative predictive value remained high in prediction models at alternative tuberculosis prevalence values (appendix 3 p 16). However, positive predictive value was 40·1% (95% CI 36·8–44·1; figure 3) in the study population, and was as low as 11·3% (95% CI 8·3–14·8) in prediction models with tuberculosis prevalence of 5%.

For a hypothetical cohort of 1000 people with presumed pulmonary tuberculosis and an underlying tuberculosis prevalence of 10%, using Xpert HR as a triage test followed by sputum testing if Xpert HR is positive (TB Score less than or equal to −1·25), ten (10%) of 100 people with tuberculosis would be missed. Of 900 people without tuberculosis, 563 would be correctly identified (as being without tuberculosis) by Xpert HR, and 337 would have positive Xpert HR results and would therefore need to undergo confirmatory sputum testing (appendix 3 p 17). Thus, using Xpert HR as a triage test would reduce the number of people requiring confirmatory sputum tuberculosis testing from 1000 to 427, a 57·3% (95% CI 54·2–60·4) reduction. For comparison, a triage test meeting minimum WHO target product profile accuracy targets would reduce the number of people requiring confirmatory sputum tuberculosis testing from 1000 to 360, a reduction of 64·0%. We assumed a tuberculosis prevalence of 10% in those presenting with symptoms as this is reflective of many settings and is consistent with Cochrane systematic review methodology for tuberculosis diagnostics.^16^

**Figure 4:**
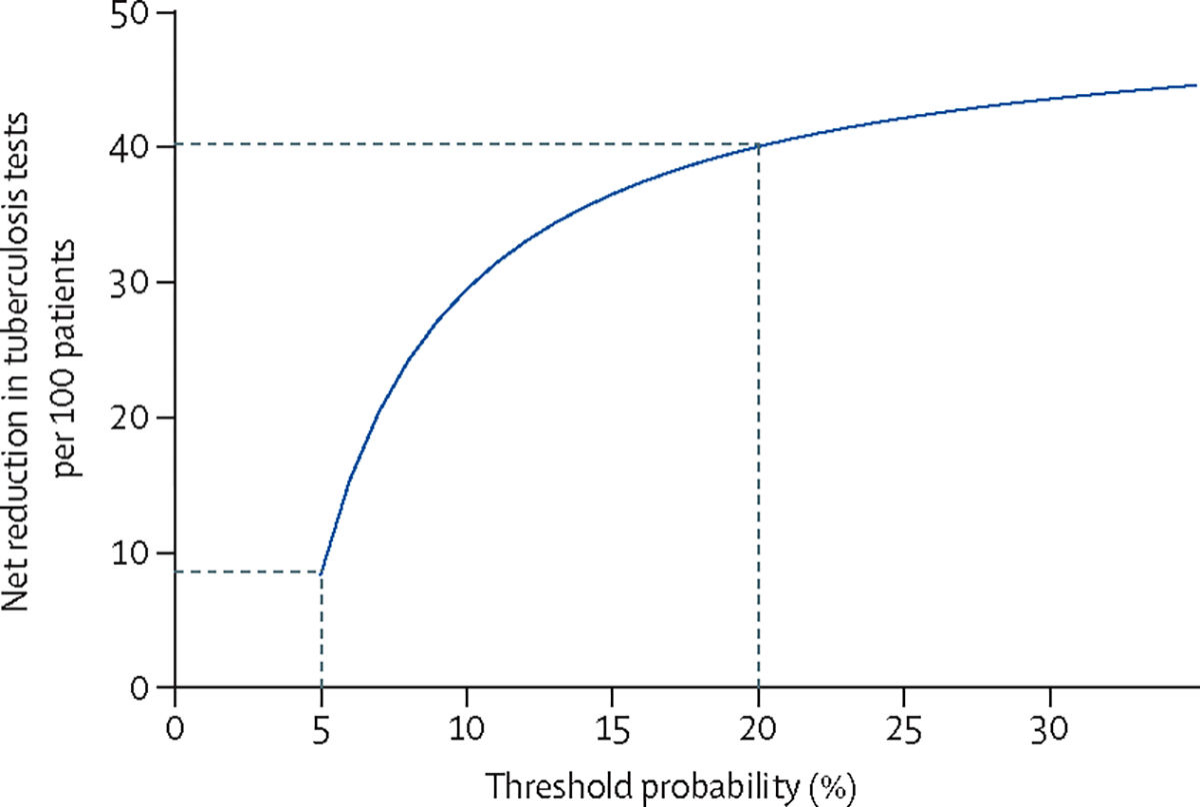
Decision curve analysis Decision curve analysis shown as net reduction in sputum-based tuberculosis tests per 100 people with presumptive tuberculosis tested with Xpert HR weighted by a range of threshold probabilities from 5 to 30%. At a 5% threshold probability, triage testing using Xpert HR would lead to a net reduction of approximately eight tuberculosis tests per 100 people. At a 20% threshold probability, triage testing using Xpert HR would lead to a net reduction of approximately 40 tuberculosis tests per 100 patients. Dashed lines indicate the 5% and 20% threshold probabilities. Xpert HR=Xpert MTB Host Response.

The decision curve analysis showed a net benefit of using Xpert HR as a triage test with a net reduction in confirmatory sputum-based tuberculosis tests needed per 100 patients across threshold probabilities of 5–30%, reflecting a range of clinician and patient risk–benefit values (figure 4). When stratifying by HIV status, net benefit was lower in people living with HIV, especially at low threshold probabilities (appendix 3 p 19).

When evaluated against the secondary reference standard of sputum Xpert Ultra only, Xpert HR had a similar AUC of 0·90 (95% CI 0·88–0·93, n=1606; appendix 3 pp 20–22). When using the same optimal TB Score cutoff value derived for the microbiological reference standard (less than or equal to −1·25), diagnostic accuracy was similar to the primary analysis: sensitivity 92·6% (95% CI 89·0–95·3) and specificity 61·6% (95% CI 58·6–63·9). However, specificity improved to 69·4% (66·8–71·9; appendix 3 p 21) when using the optimal TB Score (less than −1·45) for the sputum Xpert Ultra reference standard. Specificity was lower using the original formula for TB Score based on *KLF2* compared with the updated *TBP*-based TB Score (56·8% [95% CI 53·9–59–6] *vs* 62·6% [59·7–65·3]; p=0·0042; appendix 3 p 13).

46 (2·8%) of 1646 Xpert HR results were invalid after testing once, with three (14%) of 22 giving invalid results after repeat testing (24 were not tested a second time). Under pragmatic research conditions, 322 (96%) of 335 tested by capillary blood sampling had Xpert HR cartridges filled within 15 min of collection (median 6 min [IQR 5–9], range 0–29) as per manufacture instructions. 1322 (99·8%) of 1324 tested by venous blood sampling had Xpert HR processed within 24 h (median 1 h [IQR 0–4]), which was the timeframe recommended by the manufacturer.

## Discussion

Results of this five-country diagnostic accuracy study suggest that the Xpert HR late-prototype is the first blood-based tuberculosis assay to approximate the WHO target product profile minimum diagnostic accuracy targets (minimum sensitivity of 90% and specificity of 70%) for a tuberculosis triage test. When optimised for sensitivity at 90% or more, a specificity of 62·6% (95% CI 59·7–65·3) was achieved against the micro biological reference standard and 69·4% (66·8–71·9) against the sputum Xpert Ultra reference standard. Accuracy was similar across subgroups and settings, with the exception of about 20% lower specificity in people living with HIV (compared with those without HIV) and the need for a different cutoff to achieve similar performance in the Philippines. Xpert HR also showed a high negative predictive value across countries and subgroups while reducing the number of people requiring confirmatory sputum tuberculosis testing by more than 50%, thereby making it a potentially useful rule-out or triage test for pulmonary tuberculosis.

The Xpert HR late-prototype represents the first mRNA gene expression signature translated to a near-patient platform. To our knowledge, this is the largest prospective, multi-country diagnostic study of the Xpert HR late-prototype assay in adults. The Sweeney^6,17^ three-gene signature (comprising *GBP5*, *DUSP3*, and *KLF2*) on which the Xpert HR assay is based, was found to meet minimum WHO target product profile accuracy targets for a triage test in early case-control studies, and had the highest accuracy when prospectively compared with other candidate transcriptional signatures in South Africa.^18^ An interim analysis of a different prospective multi-country study suggested that the Xpert HR assay exceeded WHO target product profile targets when evaluated against a sputum Xpert Ultra reference standard (specificity 86% [95% CI 75–97] with sensitivity optimized at 90%).^9^ Xpert Ultra has a pooled sensitivity of 91% and specificity of 96% against culture.^16^ Our study found a lower TB Score to be associated with a higher sputum mycobacterial burden and lower BMI, indicative of more severe tuberculosis.^19^ Thus, one could postulate a different spectrum of disease explaining the somewhat lower specificity in our study. A different spectrum of disease is also likely to explain our finding of lower sensitivity in the Philippines, where Xpert Ultra positivity and semiquantitative grades were lowest. The potential need for local calibration of cutoffs might be a limitation.

When compared with the microbiological reference standard, we found specificity was lower than expected in people living with HIV, which the study by Sutherland and colleagues^9^ did not observe. Nevertheless, a difference seems plausible considering the pathophysiology of diseases: the *GBP5* gene, which codes a GTPase, is involved in interferon-induced pro-inflammatory responses and is a key gene in active tuberculosis. *GBP5* is also upregulated in viral infections such as influenza, SARS-CoV-2 and HIV.^20–22^ The *DUSP3* gene regulates inflammatory responses via control of the ERK and JNK pathways, which might also be upregulated in other diseases.^23^ Thus, the lower specificity in people living with HIV might be due to a higher prevalence of incipient or extrapulmonary tuberculosis being missed and thus being an underestimate. However, it is also possible that HIV or other infections trigger an interferon-driven immune response.^24^ Our finding of lower specificity in capillary sampling was likely to have been confounded by higher HIV prevalence among these participants.

There is a clear need for non-sputum-based triage tests to improve tuberculosis case finding while reducing the number of people who require confirmatory testing. Based on our results, using Xpert HR as a triage test for facility-based case finding would have avoided more than half of confirmatory tests, while missing only 10% of people with pulmonary tuberculosis (assuming a 10% tuberculosis prevalence). A lower tuberculosis prevalence would have led to greater reductions in confirmatory testing. Decision curve analysis showed a net benefit of using Xpert HR compared with testing all patients with confirmatory sputum tests. This is of potential benefit where the capacity or resources to perform confirmatory testing is limited, or if the cost of triage testing is less than the savings from avoided confirmatory tests.

The cost of the Xpert HR assay is currently unknown, but is likely to exceed target thresholds of US$2 per test as recommended by WHO.^3^ Cost-effectiveness modelling will be important to establish use cases and cost thresholds for the Xpert HR assay as a triage test based on the diagnostic accuracy we report.^25^ Furthermore, more detailed data on usability and acceptability will be needed to support policy recommendations.

The strengths of this study are the size of the cohort, inclusion of geographically diverse sites in Asia and Africa, and a representative sample including important comorbidities such as HIV and diabetes. We also included a robust microbiological reference standard and a pragmatic reference standard reflective of practice in tuberculosis endemic settings. This study also had important limitations. The cohort included only patients with pulmonary tuberculosis who produced sputum spontaneously or by induction, therefore our findings are not necessarily applicable to those unable to produce sputum or with extrapulmonary tuberculosis. The overall prevalence of tuberculosis was 21·9% and varied between 8·9% and 38·0%. Different disease stages might affect the sensitivity and specificity of the test. The Philippines was observed to have the lowest performance, which might be explained by a higher number of people with disease in early stages as suggested by the lowest Xpert Ultra positivity and semiquantitative grades in the Philippines. We also excluded 115 patients overall who could not be classified by the microbiological reference standard. Our reference standard might have missed some tuberculosis diagnoses, including for some patients with extrapulmonary tuberculosis, and therefore might have underestimated specificity. There is potential for Xpert HR as a diagnostic test in extrapulmonary tuberculosis, or in patients who are sputum scarce, although the limited specificity might also limit its value as a diagnostic tool.

In conclusion, Xpert HR, a novel host RNA transcriptomic signature-based test, did not meet minimum specificity targets for a non-sputum-based triage test for pulmonary tuberculosis in the context of facility-based active case finding in high tuberculosis burden settings. Although Xpert HR shows potential as a triage test that could reduce the number of confirmatory sputum tests needed, further cost-effectiveness modelling and data on acceptability and usability are needed to inform recommendations for use as a triage test for pulmonary tuberculosis.

## Supplementary Material

Supplementary appendix 1

Supplementary appendix 2

Supplementary appendix 4

Supplementary appendix 3

## Figures and Tables

**Table:** Demographic and clinical characteristics overall and by country

	Overall (n=1499)	Philippines (n=325)	Viet Nam (n=284)	South Africa (n=300)	Uganda (n=314)	India (n=276)

Sex						
Female	686 (45·8%)	180 (55·4%)	110 (38·7%)	148 (49·3%)	135 (43·0%)	113 (40·9%)
Male	813 (54·2%)	145 (44·6%)	174 (61·3%)	152 (50·7%)	179 (57·0%)	163 (59·1%)
Mean age, years	41·0 (29·0–54·0)	39·0 (25·0–52·0)	53·0 (40·0–64·0)	38·5 (31·0–48·0)	32·0 (25·0–42·0)	47·0 (35·0–59·5)
Median BMI, kg/m^2^	21·4 (19·1–25·2)	23·8 (20·2–27·5)	20·2 (18·7–21·8)	22·2 (19·3–28·4)	20·9 (19·0–24·0)	22·4 (18·9–25·9)
Cough for 2 weeks	1499 (100%)	325 (100%)	284 (100%)	300 (100%)	314 (100%)	276 (100%)
Fever	533 (35·6%)	50 (15·4%)	101 (35·6%)	107 (35·7%)	210 (66·9%)	65 (23·6%)
Weight loss	702 (46·8%)	91 (28·0%)	84 (29·6%)	192 (64·0%)	213 (67·8%)	122 (44·2%)
Night sweats	528 (35·2%)	56 (17·2%)	92 (32·4%)	162 (54·0%)	192 (61·1%)	26 (9·4%)
Previous tuberculosis diagnosis*	290 (19·3%)	43 (13·2%)	59 (20·8%)	111 (37·0%)	38 (12·1%)	39 (14·1%)
Person living with HIV†						
Yes	229 (15·3%)	3 (0·9%)	2 (0·7%)	118 (39·7%)	96 (30·6%)	10 (3·6%)
Median CD4 count, cells per μL	399 (206–667)	527·0 (184–812)	547 (497–597)	402 (206–667)	377 (211–636)	587 (220–692)
Diabetes	202 (13·5%)	42 (12·9%)	67 (23·6%)	18 (6·0%)	25 (8·0%)	50 (18·1%)
Sputum Xpert Ultra status‡ positive	297 (19·8%)	22 (6·8%)	96 (33·8%)	49 (16·3%)	106 (33·8%)	24 (8·7%)
Highest semiquantitative sputum Xpert Ultra result						
Trace§	9 (3·0%)	1 (4·5%)	0	3 (5·8%)	4 (3·7%)	1 (4·0%)
Very low	44 (14·5%)	5 (22·7%)	14 (14·6%)	10 (19·2%)	11 (10·2%)	4 (16·0%)
Low	95 (31·4%)	8 (36·4%)	38 (39·6%)	13 (25·0%)	26 (24·1%)	10 (40·0%)
Medium	76 (25·1%)	6 (27·3%)	22 (22·9%)	12 (23·1%)	29 (26·9%)	7 (28·0%)
High	79 (26·1%)	2 (9·1%)	22 (22·9%)	14 (26·9%)	38 (35·2%)	3 (12·0%)
Tuberculosis microbiological reference standard positive	329 (21·9%)	29 (8·9%)	108 (38·0%)	57 (19·0%)	109 (34·7%)	26 (9·4%)
Venous or capillary blood collection						
Capillary	286 (19·1%)	0	0	286 (95·3%)	0	0
Venous	1213 (80·9%)	325 (100%)	284 (100%)	14 (4·7%)	314 (100%)	276 (100%)

Data are n (%) or median (IQR).

* Three participants were unsure about previous tuberculosis diagnoses and treated as missing data for this variable. All participants with previous tuberculosis diagnoses had not taken tuberculosis treatment within the past 12 months.

† Three participants had an unknown HIV status.

‡ One participant was missing a sputum Xpert Ultra result, six of nine participants had only a single Xpert Ultra trace result and were therefore considered Xpert Ultra negative.

§ Five of nine participants were positive by microbiological reference standard including three participants with two trace results; four of nine participants had a single trace result with negative second Xpert Ultra and negative tuberculosis culture.

## Data Availability

Data including individual de-identified participant data and a data dictionary are available at https://data.mendeley.com/datasets/3yrg8svy8p/1.

## Research in context

### Evidence before this study

WHO has prioritised a non-sputum based triage test as a rule-out test for tuberculosis to improve diagnosis. Host blood transcriptomic signatures offer promise, and the late-prototype Xpert MTB Host Response (Xpert HR) assay is the first rapid, low complexity, automated molecular test incorporating a host response-based signature. On Sept 12, 2023, we searched PubMed for articles evaluating the performance of the Xpert HR assay for diagnosis of tuberculosis using the terms “(tuberculosis OR TB)”, “host response” AND “(Xpert OR GeneXpert OR cartridge)” without any language or date restrictions. The search yielded 154 results, of which three studies evaluated the accuracy of the Xpert HR assay. An interim analysis of a multisite study found 86% specificity when optimised for 90% sensitivity; however, this was based on a small number of participants. Other studies used retrospective testing from stored samples or used case-control designs.

### Added value of this study

To our knowledge, this is the largest prospective multicountry diagnostic study of the Xpert HR late-prototype assay in adults, which included peripheral health-care settings with high HIV and diabetes burden and allowed well powered comparisons across the different settings and subgroups. We assessed performance against a robust microbiological reference standard and a pragmatic reference standard based on Xpert MTB/RIF Ultra testing of sputum.

### Implications of all the available evidence

The Xpert HR assay, a first-in-class host RNA response-based test, did not meet WHO minimum specificity targets for a non-sputum triage test for pulmonary tuberculosis. However, the test did show promise as a rule-out test, with high negative predictive values across settings and subgroups. It showed the potential to reduce the number of confirmatory sputum tests needed. Further data on cost-effectiveness and acceptability and usability are needed to inform recommendations for use as a triage test for pulmonary tuberculosis.
