# Supplementary material for: Evaluation of the Xpert MTB Host Response assay for the triage of patients with presumed pulmonary tuberculosis: a prospective diagnostic accuracy study in Viet Nam, India, the Philippines, Uganda, and South Africa
Source: Lancet Glob Health. Author manuscript; Available in PMC 2024 Apr 26. (PMC11046618; doi:10.1016/S2214-109X(23)00541-7)
Supplement: Supplementary appendix 1 [file NIHMS1982734-supplement-Supplementary_appendix_1.pdf]

THE LANCET
Global Health

Supplementary appendix 1

This translation in Vietnamese was submitted by the authors and we reproduce it as supplied. It has not been peer reviewed. *The Lancet*'s editorial processes have only been applied to the original in English, which should serve as reference for this manuscript.

Bản dịch sang tiếng Việt này do các tác giả gửi và chúng tôi sao chép lại như đã cung cấp. Bản dịch này không qua phản biện. Quy trình biên tập của The Lancet chỉ áp dụng cho bản gốc bằng tiếng Anh, và độc giả chỉ nên dùng bản gốc tiếng Anh làm tài liệu tham khảo.

Supplement to: Gupta-Wright A, Ha H, Abdulgadar S, et al. Evaluation of the Xpert MTB Host Response assay for the triage of patients with presumed pulmonary tuberculosis: a prospective diagnostic accuracy study in Viet Nam, India, the Philippines, Uganda, and South Africa. *Lancet Glob Health* 2024; **12:** e226–34.

Tóm tắt

Đặt vấn đề

Xét nghiệm sàng lọc lao không sử dụng bệnh phẩm đờm là một ưu tiên trong việc chấm dứt bệnh lao. Nghiên cứu nhằm đánh giá mức độ chính xác trong chẩn đoán lao của xét nghiệm phản ứng vật chủ (Host Response) sử dụng bệnh phẩm máu, gọi là Xpert HR.

Phương pháp

Chúng tôi đã thực hiện nghiên cứu tiến cứu đánh giá độ chính xác trong chẩn đoán lao với những bệnh nhân ngoại trú nghi lao đến khám tại năm Quốc gia: Việt Nam, Ấn Độ, Philipin, Uganda, và Nam Phi. Người tham gia đủ tiêu chuẩn có tuổi 18 trở lên, và ho kéo dài ít nhất 2 tuần. Chúng tôi loại trừ những người đã điều trị lao trong 12 tháng trước đó hoặc không đồng ý tham gia. Xét nghiệm Xpert HR thực hiện trên máu mao mạch hoặc tĩnh mạch. Xét nghiệm tham chiếu là Xpert MTB/RIF Ultra đờm và nuôi cấy tìm vi khuẩn lao. Chúng tôi đã thực hiện phân tích đường cong ROC để xác định điểm cắt tối ưu cho Xpert HR nhằm đạt được độ nhạy ≥90% trong khi tối đa hóa độ đặc hiệu của xét nghiệm, sau đó tính toán độ chính xác trong chẩn đoán sử dụng điểm cắt này. Nghiên cứu đã đăng ký tại ClinicalTrials.gov, NCT04923958.

Kết quả

Từ 13 tháng 7 năm 2021 đến 15 tháng 8 năm 2022, trong 2046 người lớn có ho ít nhất 2 tuần, 1499 người (686 [45,8%] nữ; và 813 [54,2%] nam) có kết quả Xpert HR hợp lệ và kết quả xét nghiệm tham chiếu. 329 (21,9%) được chẩn đoán có bằng chứng vi khuẩn. Xpert HR có diện tích dưới đường cong ROC là 0,89 (KTC 95%: 0,86-0,91). Điểm cắt tối ưu nhỏ hơn hoặc bằng 1,25, cho độ nhạy 90,3% (KTC 95%: 86,5-93,3) và độ đặc hiệu 62,6% (KTC 95%: 59,7-65,3). Độ nhạy tương tự nhau giữa các quốc gia, theo giới tính và phân nhóm, mặc dù độ đặc hiệu thấp hơn ở nhóm người nhiễm HIV (45,1%, KTC 95%: 37,8-52,6) so với người không nhiễm (65,9%, 62,8-68,8; khác biệt 20,8%, KTC 95%: 13,0-28,6; p<0,0001). Xpert HR có giá trị dự đoán âm tính cao (95,8%, KTC 95%: 94,1-97,1), nhưng giá trị dự đoán dương tính chỉ là 40,1% (KTC 95%: 36,8-44,1). Sử dụng Xpert HR làm xét nghiệm sàng lọc có thể giảm được 57,3% (KTC 95%: 54,2-60,4) việc sử dụng xét nghiệm khẳng định lao bằng đờm.

Diễn giải

Xpert HR chưa đáp ứng được mục tiêu về độ đặc hiệu tối thiểu của Tổ chức Y tế Thế giới (WHO) đối với xét nghiệm sàng lọc lao phổi không dùng bệnh phẩm đờm. Mặc dù có tiềm năng là xét nghiệm 'loại trừ' bệnh lao qua việc giảm sử dụng các xét nghiệm khẳng định bằng đờm, các mô hình chi phí hiệu quả và các dữ liệu về khả năng chấp nhận và sử dụng xét nghiệm cần được thu thập nhằm cung cấp thông tin khuyến nghị chính sách.

Tài trợ

Nghiên cứu được tài trợ bởi Viện Dị ứng và Bệnh Truyền nhiễm Quốc gia của Viện Sức khoẻ Quốc gia Hoa Kỳ.
