# Supplementary material for: Evaluation of the Xpert MTB Host Response assay for the triage of patients with presumed pulmonary tuberculosis: a prospective diagnostic accuracy study in Viet Nam, India, the Philippines, Uganda, and South Africa
Source: Lancet Glob Health. Author manuscript; Available in PMC 2024 Apr 26. (PMC11046618; doi:10.1016/S2214-109X(23)00541-7)
Supplement: Supplementary appendix 2 [file NIHMS1982734-supplement-Supplementary_appendix_2.pdf]

THE LANCET
Global Health

Supplementary appendix 2

This translation in Tagalog was submitted by the authors and we reproduce it as supplied. It has not been peer reviewed. *The Lancet*'s editorial processes have only been applied to the original in English, which should serve as reference for this manuscript.

Ang pagsasaling ito sa Tagalog ay isinumite ng mga may-akda at aming muling ginawa ayon sa ibinigay nila. Hindi ito nasuri ng mga kapantay na propesyonal. Ang mga proseso ng editoryal ng Lancet ay inilapat lamang sa orihinal na Ingles, at dapat magsilbing sanggunian para sa manuskrito na ito.

Supplement to: Gupta-Wright A, Ha H, Abdulgadar S, et al. Evaluation of the Xpert MTB Host Response assay for the triage of patients with presumed pulmonary tuberculosis: a prospective diagnostic accuracy study in Viet Nam, India, the Philippines, Uganda, and South Africa. *Lancet Glob Health* 2024; **12:** e226–34.

Pagsusuri sa paraan ng pagsisiyasat (assay) ng* Tugon ng organismo sa impeksyon ng pathogen (*Host Response*) ng Xpert MTB *para sa pag-uuri (triage) ng mga pasyente na ipinapalagay na may pulmonary tuberculosis: isang maramihang-site na inaasahang pag-aaral ng katumpakan ng dayagnostiko (multi-site prospective diagnostic accuracy study)

Kasaysayan sa likod ng pag-aaral
Ang hindi nakabatay-sa-plema (*non sputum-based*) na mga pagsubok sa pag-uuri (*triage tests*) para sa tuberkulosis ay isang prayoridad para sa pagtatapos ng tuberkulosis. Sinuri namin ang katumpakan ng dayagnostiko ng huling-prototype na *Xpert MTB Host Response (Xpert HR)* na batay-sa-dugong paraan ng pagsisiyasat (*assay*).

Mga Pamamaraan
Nagsagawa kami ng inaasahang pag-aaral ng katumpakan ng dayagnostiko sa mga pasyenteng tumanggap ng paggamot pero hindi naospital (*outpatient*) na ipinapalagay na may tuberculosis sa Viet Nam, India, Pilipinas, Uganda at South Africa. Ang karapat-dapat na mga kalahok ay may edad na 18 taon o mas matanda at nag-ulat ng ubo sa loob ng hindi bababa sa 2 linggo. Hindi isinama ang mga tumatanggap ng paggamot sa tuberkulosis sa naunang 12 buwan o hindi gustong sumang-ayon. Ang *Xpert HR* ay isinagawa sa maliliit na ugat (*capillary*) o sa *venous blood*. Kasama sa sangguniang pamantayan ng pagsubok ang *sputum Xpert MTB/RIF Ultra* at *mycobacterial culture*. Nagsagawa kami ng pag-analisa sa *receiver operating characteristic* (*ROC*) upang matukoy ang pinakamainam na punto-ng-hangganan (*cut-point*) para sa *Xpert HR* upang makamit ang porsyento ng mga taong may sakit na natukoy nang tama ng pagsusuri (*target sensitivity*) na ≥90% habang itinotodo ang porsyento ng mga taong walang sakit na ibinukod nang tama ng pagsusuri (*specificity*), pagkatapos ay kinakalkula ang katumpakan ng dayagnostiko gamit ang punto-ng-hangganan na ito.

Mga Natuklasan
Sa pagitan ng Hulyo 13, 2021 at Agosto 15, 2022, 2046 na matatanda at may ubo na hindi bababa sa 2 linggo ang , kung saan 1499 na matatanda (686[45.8%] babae at 813 [54.2%] lalaki) nagkaroon ng wastong *Xpert HR* at sangguniang pamantayan na mga resulta. 329 (21.9%) ang nakumpirmang may tuberculosis kung saan ang biyolohikal na ispesimen ay positibo (*microbiologically-confirmed tuberculosis)*. Ang *Xpert HR* ay nagkaroon ng lugar sa ilalim ng ROC curve na 0.89 (95%CI 0.86-0.91). Ang pinakamainam na *cut-point* ay ≤-1.25, na nagbibigay ng 90.3% (95%CI 86.5-93.3; 297 of 329) na *sensitivity* at 62.6% (95%CI 59.7-65.3; 732 of 1170) na *specificity*. Ang *sensitivity* ay magkapareho sa mga bansa, ayon sa kasarian at mga *subgroup*, bagaman mas mababa ang *specificity* sa mga taong nabubuhay na may HIV (45.1% , 95% CI 37.8-52.6) kumpara sa mga nabubuhay na walang HIV (65.9%, 95% CI 62.8-68.8; difference of 20.8%, 95% CI 13.0-28.6; p<0.0001). Ang *Xpert HR* ay may mataas na negatibong hinuhulaang halaga (*predictive value*) (95.8%, 95%CI 94.1-97.1), pero ang positibong hinuhulaang halaga ay 40.1% lamang (95%CI 36.8-44.1). Ang paggamit ng *Xpert HR* bilang isang pagsubok sa pag-uuri (*triage test*) ay maaaring makabawas sa pagsusuri sa pagkumpirma ng *sputum* ng 57.3% (95% CI 54.2-60.4).

Interpretasyon

Hindi nakamit ng *Xpert HR* ang pinakamababang *specificity target* ng WHO para sa isang hindi-nakabatay-sa-plema na mga pagsubok sa pag-uuri para sa *pulmonary tuberculosis*. Sa kabila ng pangako bilang isang 'nagbubukod (*rule-out)*' na pagsubok na maaaring makabawas sa pagsusuri sa pagkumpirma ng *sputum*, higit pang disenyo ng pagiging-epektibo-ng-gastos at datos sa pagiging katanggap-tanggap at kakayahang magamit ang kinakailangan para ipaalam ang mga rekomendasyon sa patakaran.

Pagpopondo
National Institute of Allergy and Infectious Diseases ng *US National Institutes of Health* – (U01AI152087). Ang mga *cartridge* ng Xpert HR ay ipinagkaloob ng Cepheid.
