# Supplementary material for: Evaluation of the Xpert MTB Host Response assay for the triage of patients with presumed pulmonary tuberculosis: a prospective diagnostic accuracy study in Viet Nam, India, the Philippines, Uganda, and South Africa
Source: Lancet Glob Health. Author manuscript; Available in PMC 2024 Apr 26. (PMC11046618; doi:10.1016/S2214-109X(23)00541-7)
Supplement: Supplementary appendix 4 [file NIHMS1982734-supplement-Supplementary_appendix_4.pdf]

THE LANCET
Global Health

Supplementary appendix 4

This Equitable Partnership Declaration (EPD) was submitted by the authors, and we reproduce it as supplied. It has not been peer reviewed. *The Lancet*'s editorial processes have not been applied to the EPD.

Supplement to: Gupta-Wright A, Ha H, Abdulgadar S, et al. Evaluation of the Xpert MTB Host Response assay for the triage of patients with presumed pulmonary tuberculosis: a prospective diagnostic accuracy study in Viet Nam, India, the Philippines, Uganda, and South Africa. *Lancet Glob Health* 2024; **12:** e226–34.

Equitable Partnership Declaration questions

Researcher considerations

1. Please detail the involvement that researchers who are based in the region(s) of study had during a) study design; b) clinical study processes, such as processing blood samples, prescribing medication, or patient recruitment; c) data interpretation; and d) manuscript preparation, commenting on all aspects. If they were not involved in any of these aspects, please explain why.

   This question is intended for international partnerships; if all your authors are based in the area of study, this question is not applicable.

   This should include a thorough description of their leadership role(s) in the study. Are local researchers named in the author list or the acknowledgements, or are they not mentioned at all (and, if not, why)? Please also describe the involvement of early career researchers based in the location of the study. Some of this information might be repeated from the Contributors section in the manuscript. Note: we adhere to ICMJE authorship criteria when deciding who should be named on a paper.

**a) Study design:**<br>All the PIs from the country sites of the study had detailed involvement in the study design, including acquiring the funding. They take an important leadership role for the local study sites, but also a leadership role for the project as part of the steering committee for the R2D2 study network. They are all named as co-authors for this manuscript.
**b) Clinical study processes:**<br>PIs and other research staff from each study site were pivotal to the study processes overall and locally at their sites. They all helped co-design study procedures and data collection tools, including appropriate local adaptation. The key local research staff that coordinated the study, and contributed to clinical and laboratory data collection and study procedures are named as authors in the author list, or individually listed under the group authorship (see appendix).
**c) Data interpretation:**<br>Data analysis and interpretation involved local study site PIs and coordinating research staff during monthly data analysis calls, as well as during manuscript preparation. This provided key insights into the data and shaped the interpretation over the whole duration of the study.
**d) Manuscript preparation:**<br>The manuscript was prepared by a writing group (those named in the author list), which included at least 2 representatives (one junior and one senior) from each local study site. This was crucial for appropriate contextual understanding and interpretation of the findings.

2. Were the data used in your study collected by authors named on the paper, or have they been extracted from a source such as a national survey? ie, is this a secondary analysis of data that were not collected by the authors of this paper. If the authors of this paper were not involved in data collection, how were data interpreted with sufficient contextual knowledge?

The Lancet Global Health *believe contextual understanding is crucial for informed data analysis and interpretation.*

The data used in this study have been collected by named authors.

3. How was funding used to remunerate and enhance the skills of researchers and institutions based in the area(s) of study? And how was funding used to improve research infrastructure in the area of study?

   Potentially effective investments into long-term skills and opportunities within institutions could include training or mentorship in analytical techniques and manuscript writing, opportunities to lead all or specific aspects of the study, financial remuneration rather than requiring volunteers, and other professional development and educational opportunities.

   Improvements to research infrastructure could be funding of extended trial designs (such as platform trials) and use of master protocols to enable these designs, establishment of long-term contracts for research staff, building research facilities, and local control of funding allocation.

**Skills:**<br>Study teams at local sites were provided with mentoring opportunities with scientists from UCSF, UKHD and broader team. This includes a capacity building seminar series delivered by the R2D2 team, and opportunities for training in data analysis and statistical methodology. Funding is used for training and progression of local research staff wherever possible, and study procedures are utilised for local capacity development at every opportunity.
**Research infrastructure:**<br>The R2D2 study has helped build on existing research infrastructure at each site to establish a clinical trial network capable of high-quality TB research in high burden settings. The master R2D2 protocol has been leveraged for additional funding from international funders for further TB diagnostic studies that will allow for extended funding for sites, supporting long-term contracts for staff, improving facilities and obtaining local research funding.

4. How did you safeguard the researchers who implemented the study?

   Please describe how you guaranteed safe working conditions for study staff, including provision of appropriate personal protective equipment, protection from violence, and prevention of overworking.

The R2D2 study sites were led by local PIs who ensured the safeguarding of study staff. R2D2 funded appropriate PPE for local staff.

<u>Benefits to the communities and regions of study</u>

5. How does the study address the research and policy priorities of its location?

How were the local priorities determined and then used to inform the research question? Who decided which priorities to take forward? Which elements of the study address those priorities?

TB continues to be a major global health challenge. All the R2D2 study sites are in high TB, TB and HIV co-infection or Drug Resistant-TB settings. Local PIs were involved in co-designing the study, and therefore were able to take forward their priorities too. Modelling, cost-effectiveness and assessing usability and feasibility is a key part of the study design, and reflects local priorities. Being able to quickly and accurately diagnose patients with TB, reducing over and under-diagnosis, and doing so in way that is acceptable to patients and healthcare workers is a key local priority and at the heart of the R2D2 study. Results are also communicated via the local PIs to local policy makers.

6. How will research products be shared in the community of study?

For instance, will you be providing written or oral layperson summaries for non-academic information sharing? Will study data be made available to institutions in the region(s) of study? The Lancet Global Health *encourages authors to translate the summary (abstract) into relevant languages after paper editing; do you intend to translate your summary?*

The study dissemination plan includes written layperson summaries for dissemination to patient and civil society groups. These will also be provided in local languages where appropriate. Anonymised study data will published in a data repository upon publication of manuscript. Other data related to the study is available upon request. The Philippines and Vietnam teams are able to translate the summary into local languages. Due to the range of local languages spoken and other challenges, this will not be possible at the other sites.

7. How were individuals, communities, and environments protected from harm?

a) How did you ensure that sensitive patient data was handled safely and respectfully? Was there any potential for stigma or discrimination against participants arising from any of the procedures or outcomes of the study?

Data was collected sensitively and in keeping with appropriate local culture, as led by the local study site PI and their research team. All data was collected electronically into a password-protected REDCap database. Data minimization principles were applied. Access was restricted to only those who needed access.

b) Might any of the tests be experienced as invasive or culturally insensitive?

Not to our knowledge

c) *How did you determine that work was sensitive to traditions, restrictions, and considerations of all cultural and religious groups in the study population?*

The study procedures were adapted by the local PIs and site teams who were familiar with local traditions, restrictions, and cultural and religious groups. This allowed us to be as sensitive as possible, and safeguarding the interests of our participants.

d) *Were biowaste and radioactive waste disposed of in accordance with local laws?*

Yes, as specified in study procedures

e) *Were any structures built that would have impacted members of the community or the environment (such as handwashing facilities in a public space)? If so, how did you ensure that you had appropriate community buy-in?*

Not applicable

f) *How might the study have impacted existing health-care resources (such as staff workloads, use of equipment that is typically employed elsewhere, or reallocation of public funds)?*

The study was designed to have minimal impact on existing health-care resources. Some routine diagnostics may have been done by the study rather than by the health-care system, thus potentially reducing resources used locally.

8. Finally, please provide the title (eg, Dr/Prof, Mr/Mrs/Ms/Mx), name, and email address of an author who can be contacted about this statement. This can be the corresponding author.

Name: Dr Ankur Gupta-Wright
Email: agupta@uni-heidelberg.de
