# Supplementary material for: Evaluation of the Xpert MTB Host Response assay for the triage of patients with presumed pulmonary tuberculosis: a prospective diagnostic accuracy study in Viet Nam, India, the Philippines, Uganda, and South Africa
Source: Lancet Glob Health. Author manuscript; Available in PMC 2024 Apr 26. (PMC11046618; doi:10.1016/S2214-109X(23)00541-7)
Supplement: Supplementary appendix 3 [file NIHMS1982734-supplement-Supplementary_appendix_3.pdf]

THE LANCET
Global Health

Supplementary appendix 3

This appendix formed part of the original submission and has been peer reviewed. We post it as supplied by the authors.

Supplement to: Gupta-Wright A, Ha H, Abdulgadar S, et al. Evaluation of the Xpert MTB Host Response assay for the triage of patients with presumed pulmonary tuberculosis: a prospective diagnostic accuracy study in Viet Nam, India, the Philippines, Uganda, and South Africa. *Lancet Glob Health* 2024; **12:** e226–34.

Appendix

Details of additional contributors from the R2D2 TB Network 2

Supplementary methods 5
Table S1. R2D2 TB Network study enrolment sites and ethics committees 5
Table S2. Procedure summary during participant enrolment and follow up 6
Reasons for South Africa using capillary blood 8
Details of reference standards 8

Supplementary results 10
Table S3: Demographical and clinical characteristics overall and by Microbiological Reference Standard (MRS) result 11
Figure S1. Histogram of Xpert HR TB Score and its components 12
Figure S2. Xpert TB HR Receiver Operating Characteristic (ROC) curve using previous calculation against microbiologic reference standard. 13
Table S4: Univariable regression between TB Score and other variables 14
Table S5: Area Under the Receiver Operating Characteristic (ROC) curve, overall and by subgroups 14
Table S6: Diagnostic accuracy of Xpert-HR by blood sampling method, stratified by HIV-status 15
Figure S3: Positive and negative predictive values for the Xpert-HR and WHO minimum Target Product Profile accuracy for a range of TB prevalence 16
Figure S4: Numbers needing sputum testing based on a hypothetical cohort of 1000 patients with presumed TB and 10% TB prevalence 17
Table S9: Diagnostic Accuracy for the Xpert-HR based on Microbiological Reference Standard overall and by subgroup 18
Figure S5: Decision Curve Analysis stratified by HIV status 19
Table S8: Demographical and clinical characteristics overall and by country for secondary analysis using Sputum Xpert Reference Standard 20
Table S9: Diagnostic Accuracy for the Xpert-HR based on Sputum Xpert Reference Standard overall and by subgroup 21
Figure S5. Xpert TB HR Receiver Operating Characteristic (ROC) curve analysis against sputum Xpert reference standard. 22

STARD checklist 23

Details of additional contributors from the R2D2 TB Network

First name	Surname	Affiliation
**India**		
Balamugesh	Thangakunam	Christian Medical College, Vellore
Deepa	Shankar	Christian Medical College, Vellore
Vinita	Ernest	Christian Medical College, Vellore
Flavita	John	Christian Medical College, Vellore
Bharath	Karthikeyan	Christian Medical College, Vellore
Reena	Sekar	Christian Medical College, Vellore
Divya	Mangal	Christian Medical College, Vellore
Sai	Vijayasree	Christian Medical College, Vellore
Swetha	Sankar	Christian Medical College, Vellore
Mary	Shibiya	Christian Medical College, Vellore
Priyadarshini	Gajendran	Christian Medical College, Vellore
Shanmugasundaram	Elango	Christian Medical College, Vellore
Rajasekar	Sekar	Christian Medical College, Vellore
**Philippines**		
Jared	Almonte	De La Salle Medical and Health Sciences Institute
Ramon	Basilio	National TB Reference Laboratory, Research Institute for Tropical Medicine, Department of Health
Asella Ruvijean	Cariaga	De La Salle Medical and Health Sciences Institute
Raul	Destura	National Institutes of Health, University of the Philippines Manila
Victoria	Dalay	De La Salle Medical and Health Sciences Institute
Karlo	Dayawon	De La Salle Medical and Health Sciences Institute
Darecil	Gelina	De La Salle Medical and Health Sciences Institute
Joseph Aldwin	Goleña	De La Salle Medical and Health Sciences Institute
Maria Marissa	Golla	De La Salle Medical and Health Sciences Institute
Gidalthi Jonathan	Ilagan	De La Salle Medical and Health Sciences Institute
Dodge	Lim	National TB Reference Laboratory, Research Institute for Tropical Medicine, Department of Health
Angelita	Pabruada	De La Salle Medical and Health Sciences Institute
Annalyn	Reyes	De La Salle Medical and Health Sciences Institute
Roeus Vincent Arjay G.	Reyes	National TB Reference Laboratory, Research Institute for Tropical Medicine, Department of Health
Maricef	Tonquin	De La Salle Medical and Health Sciences Institute
**South Africa**		
Brigitta	Derendinger	Stellenbosch University
Megan	Hendrikse	Stellenbosch University
Anna	Okunola	Stellenbosch University
Zaida	Palmer	Stellenbosch University
**Uganda**		
Alfred	Andama	Makerere University College of Health Sciences, Department of Medicine<br>Kampala, Uganda
Esther	Kisakye	World Alliance for Lung and Intensive Care Medicine in Uganda (WALIMU)
Job	Mukwatamundu	World Alliance for Lung and Intensive Care Medicine in Uganda (WALIMU)

First name	Surname	Affiliation
Sandra	Mwebe	World Alliance for Lung and Intensive Care Medicine in Uganda (WALIMU)
Martha	Nakaye	World Alliance for Lung and Intensive Care Medicine in Uganda (WALIMU)
William	Worodria	Makerere University College of Health Sciences, Department of Medicine<br>Kampala, Uganda
Justine	Nyawere	World Alliance for Lung and Intensive Care Medicine in Uganda (WALIMU)
Alice	Bukirwa	World Alliance for Lung and Intensive Care Medicine in Uganda (WALIMU)
Wilson	Mangeni	World Alliance for Lung and Intensive Care Medicine in Uganda (WALIMU)
John Baptist	Ssonko	World Alliance for Lung and Intensive Care Medicine in Uganda (WALIMU)
Annet	Nakaweesa	World Alliance for Lung and Intensive Care Medicine in Uganda (WALIMU)
Irene	Nassuna	World Alliance for Lung and Intensive Care Medicine in Uganda (WALIMU)
Irene	Nekesa	World Alliance for Lung and Intensive Care Medicine in Uganda (WALIMU)
David	Katumba	World Alliance for Lung and Intensive Care Medicine in Uganda (WALIMU)
Lucy	Asege	World Alliance for Lung and Intensive Care Medicine in Uganda (WALIMU)
Talemwa	Nalugwa	World Alliance for Lung and Intensive Care Medicine in Uganda (WALIMU)
**Vietnam**		
Hai	Dang	Vietnam National Tuberculosis Program-University of California San Francisco Research Collaboration Unit, Hanoi, Vietnam
Luong	Dinh	Vietnam National Lung Hospital
Thien	Doan	Hanoi Lung Hospital, Hanoi, Vietnam
Hang	Do	Hanoi Lung Hospital, Hanoi, Vietnam
Tam	Do	Hanoi Lung Hospital, Hanoi, Vietnam
Hien	Le	Vietnam National Tuberculosis Program-University of California San Francisco Research Collaboration Unit, Center for Promotion of Advancement of Society, Hanoi, Vietnam
Nguyet	Le	National TB reference Lab/ Vietnam National Lung Hospital, Hanoi, Vietnam
Anh	Nguyen	Hanoi Lung Hospital, Hanoi, Vietnam
Dong	Nguyen	Hanoi Lung Hospital, Hanoi, Vietnam
Hanh	Nguyen	Vietnam National Tuberculosis Program-University of California San Francisco Research Collaboration Unit, Center for Promotion of Advancement of Society, Hanoi, Vietnam
Hoang	Nguyen	Hanoi Lung Hospital, Hanoi, Vietnam
Thanh	Nguyen	Hanoi Lung Hospital, Hanoi, Vietnam
Nam	Pham	Vietnam National Tuberculosis Program-University of California San Francisco Research Collaboration Unit, Hanoi Lung Hospital, Hanoi, Vietnam
Thuong	Pham	Hanoi Lung Hospital, Hanoi, Vietnam

First name	Surname	Affiliation
Ha	Phan	Vietnam National Tuberculosis Program-University of California San Francisco Research Collaboration Unit, Center for Promotion of Advancement of Society, Hanoi, Vietnam
Trang	Trinh	Vietnam National Tuberculosis Program-University of California San Francisco Research Collaboration Unit, Center for Promotion of Advancement of Society, Hanoi, Vietnam
**USA**		
Robert	Castro	University of California San Francisco
Catherine	Cook	University of California San Francisco
Tessa	Mochizuki	University of California San Francisco
Midori	Kato-Maeda	University of California San Francisco
Ruvandhi	Nathavitharana	Beth Israel Deaconess Medical Center<br>Harvard Medical School
Kevin	Nolan	University of California San Francisco
Kinari	Shah	University of California San Francisco
Swati	Sudarsan	University of California San Francisco
Christina	Yoon	University of California San Francisco
**Germany**		
Ioana-Diana	Olaru	Heidelberg University Hospital, Heidelberg, Germany
Maria del Mar	Castro Noriega	Heidelberg University Hospital, Heidelberg, Germany
Seda	Yerlikaya	Heidelberg University Hospital, Heidelberg, Germany

Supplementary methods

Table S1. R2D2 TB Network study enrolment sites and ethics committees

City, Country	Enrollment sites	Ethics Committee*
Vellore, India	CMC Pulmonary Outpatient Department, Primary care clinics in Vellore (Shalom/LCC, Chittor, CHAD) and Chitoor (CMC satellite campus)	Christian Medical College Institutional Review Board (13256)
Hanoi, Vietnam	Outpatient departments, Hanoi Lung Hospital	Ministry of Health Ethical Committee for National Biological Medical Research (94/CN-HĐĐĐ); National Lung Hospital Ethical Committee for Biological Medical Research (566/2020/NCKH); Hanoi Lung Hospital Science and Technology Initiative Committee (22/BVPHN)
Dasmariñas City, Philippines	Community-based screening in Dasmariñas City and nearby municipalities, outpatient clinics in Dasmariñas City	De La Salle Health Sciences Institute Independent Ethics Committee (2020-33-02-A)
Cape Town, South Africa	Scottsdene and Wallacedene primary care clinics; Brooklyn Chest Hospital; Khayelitsha District Health Center; Kraaifontein Community Health Clinic	Stellenbosch University Health Research Ethics Committee (M20/07/020)
Kampala, Uganda	Mulago Outpatient Department, Kisenyi Health Center,	Makerere University, College of Health Sciences, School of Medicine, Research Ethics Committee (2020-182)

* The study was additionally approved by the University of California San Francisco Institutional Review Board (20-32670), and the University of Heidelberg Ethics Committee of the Medical Faculty (S-539/2020).

Table S2. Procedure summary during participant enrolment and follow up

LEGEND. X: applies to all participants; Y: applies to people without a known TB diagnosis

Procedure	Enrolment		Follow- up Visit 1[1]	Notes
	Day 1	Day 2		
**Demographic/Clinical questionnaire**	X		Y	
**TB symptom assessment**	X		Y	
**Chest X-ray**	Y		Y	• All CXRs undergo digitization
**Finger prick blood collection**	X			• All participants: HIV, Hemoglobin A1c (in some sites this is done using blood collected from venipuncture in a central lab)<br>• novel blood tests
**Venipuncture[4]**	Y			• HbA1c if done in a central lab<br>• CD4 cell count (if HIV-positive)<br>• novel blood tests

**Spot sputum[4] collection**	X		Y	• Participants without known TB diagnosis: Xpert Ultra x 1, Smear microscopy and MGIT culture x 2, storage. Sputum collection will be repeated on Day 2 if initial Xpert Ultra results are indeterminate or trace-positive.<br>• Participants with RIF-resistance by Xpert: MGIT culture, DNA extraction, novel rDST assays, storage<br>• Sputum induced if participants are unable to expectorate
**Urine collection[4]**	Y			• Urine Xpert Ultra conducted on HIV-positive participants only then discontinued part-way through the study due to low yield.
**Other TB testing per clinician discretion**	X	X	X	• Results of any additional mycobacterial culture or Xpert testing from other samples (e.g. pleural fluid, tissue biopsy, etc.) requested by clinicians recorded

1 Follow-up visit 1 conducted at 3-months if the participant has a negative/indeterminate sputum Xpert Ultra result at baseline AND no positive culture

2 For all participants without confirmed TB but a positive novel TB triage or diagnostic test result, trained study staff collect 1 follow-up spot sputum specimen for Xpert Ultra (x1) and mycobacterial culture (1 liquid, 1 solid) and perform a CXR.

3 All left-over samples stored.

Reasons for South Africa using capillary blood

Cepheid instructs that blood may be collected for Xpert TB Host Response using either capillary sampling or venipuncture (phlebotomy) sampling. If capillary sampling is used, blood must not be held in the minivette for more than 15 minutes due to risk of clogging. If using venipuncture, an EDTA blood collection tube can be stored at 2-8 °C up to 24 h before testing. Due to onsite logistical constraints, South Africa was the only site able to conduct the test immediately following blood collection, allowing for capillary sampling.

Details of reference standards

<u>Primary: Microbiologic reference standard (MRS)</u>
The primary reference standard for all novel tests will be a microbiologic reference standard defined by Xpert Ultra for sputum, Xpert Ultra for urine, and sputum MGIT culture. The MRS is based only on baseline test results and does not incorporate any results from follow-up visits.

- TB positive will include participants with:
  - A positive result on sputum Xpert Ultra (grade very low, low, medium, high) **<u>OR</u>**
  - A positive culture in MGIT **<u>OR</u>**
  - A positive culture in solid culture media (e.g. 7H10 or LJ agar*) **<u>OR</u>**
  - Two trace Xpert Ultra results on sputum
- TB negative will include participants with:
  - No positive result (sputum Xpert Ultra, culture) **<u>AND EITHER</u>**
    - 2 negative cultures in MGIT **<u>OR</u>**
    - 2 negative cultures in solid media (e.g. 7H10 or LJ agar*) **<u>OR</u>**
    - 1 negative culture in MGIT and 1 negative in solid media (e.g. 7H10 or LJ agar*)†
- Indeterminate will include participants with:
  - No positive result (sputum Xpert Ultra, urine Xpert Ultra, culture), **<u>AND</u>**
  - <2 trace Ultra results on sputum and/or urine **<u>AND</u>**
  - <2 negative cultures due to contamination or pending results.

* Cultures on solid media will not routinely be done but may be done when MGIT is not available.

† The initial protocol in India included only one sputum culture from August 16 to November 18 2021. Therefore, participants enrolled in India during this period are considered to be TB negative by MRS if they do not have a positive sputum Xpert result AND their single sputum culture result is negative.

<u>Secondary: Sputum Xpert Ultra Reference Standard (SXRS)</u>
Additionally, we will consider a simplified reference standard using the same definitions as the MRS but using sputum Xpert Ultra results only to classify participants as TB positive or Not TB positive.

The objective of inclusion of this reference standard is to assess sensitivity of a novel triage test in the context of a programmatic setting where only sputum Xpert Ultra is available as a confirmatory diagnostic test (*i.e.,* culture is not done as part of routine care). Sensitivity is therefore the focus of this reference standard, nevertheless specificity will be calculated for secondary analyses.

- TB positive will include participants with:
  - A positive baseline sputum Xpert Ultra result (grade very low, low, medium, high), **<u>OR</u>**

    - Two trace baseline sputum Xpert Ultra results
- TB negative will include participants with:
    - A negative baseline sputum Xpert Ultra result, **WITHOUT** any trace or positive baseline sputum Xpert Ultra results.
- TB indeterminate will include participants where neither criteria for TB positive nor TB negative are met.

Supplementary results

Indeterminate reference standard results

Of the 115 indeterminate reference standard results: 15 had two contaminated cultures; 87 had one contaminated culture and one negative culture result; 2 had one contaminated culture and one culture positive for non-tuberculous mycobacteria; 8 had one negative and one missing culture result. Of these 115, 68 had follow-up sputum culture results of which none were positive for TB.

Table S3: Demographical and clinical characteristics overall and by Microbiological Reference Standard (MRS) result

		**Overall**	MRS TB Negative	MRS TB Positive	p-value
**N**		1499	1170	329	
**Country**	**Philippines**	325 (21.7%)	296 (25.3%)	29 (8.8%)	<0.001
	**Vietnam**	284 (18.9%)	176 (15.0%)	108 (32.8%)	
	**South Africa**	300 (20.0%)	243 (20.8%)	57 (17.3%)	
	**Uganda**	314 (20.9%)	205 (17.5%)	109 (33.1%)	
	**India**	276 (18.4%)	250 (21.4%)	26 (7.9%)	
**Sex**	**Male**	813 (54.2%)	590 (50.4%)	223 (67.8%)	<0.001
	**Female**	686 (45.8%)	580 (49.6%)	106 (32.2%)	
**Age, mean (SD), years**		42.3 (15.5)	43.2 (15.4)	39.1 (15.5)	<0.001
**BMI, median (IQR)**		21.4 (19.1, 25.2)	22.5 (19.8, 26.4)	19.4 (17.8, 21.0)	<0.001
**Cough for 2 weeks**		1499 (100.0%)	1170 (100.0%)	329 (100.0%)	
**Fever**		533 (35.6%)	344 (29.4%)	189 (57.4%)	<0.001
**Weight loss**		702 (46.8%)	475 (40.6%)	227 (69.0%)	<0.001
**Night Sweats**		528 (35.2%)	358 (30.6%)	170 (51.7%)	<0.001
**Prior TB diagnosis**[a]	**Yes**	290 (19.3%)	231 (19.7%)	59 (17.9%)	0.46
**Person living with HIV**[b]	**Yes**	229 (15.3%)	184 (15.8%)	45 (13.7%)	0.37
	**Median CD4, cells/µL (IQR)**	399 (206, 667)	445.5 (240.0, 675.5)	258.5 (115.0, 529.0)	<0.001
**Diabetes**	**Yes**	202 (13.5%)	142 (12.1%)	60 (18.2%)	0.004
**Sputum Xpert status**[c]	**Negative**	1201 (80.1%)	1169 (99.9%)	32 (9.7%)	<0.001
	**Positive**	297 (19.8%)	0 (0.0%)	297 (90.3%)	
**Venous or capillary blood collection**	**Capillary**	1170 (78.1%)	231 (19.7%)	55 (16.7%)	0.22
	**Venous**	329 (21.9%)	939 (80.3%)	274 (83.3%)	

[a]3 participants were unsure about prior TB diagnoses. [b]3 participants had an unknown HIV status. [c]1 participant was missing a sputum Xpert result.
MRS microbiological reference standard. SD Standard Deviation; IQR Interquartile Range

Figure S1. Histogram of Xpert HR TB Score and its components

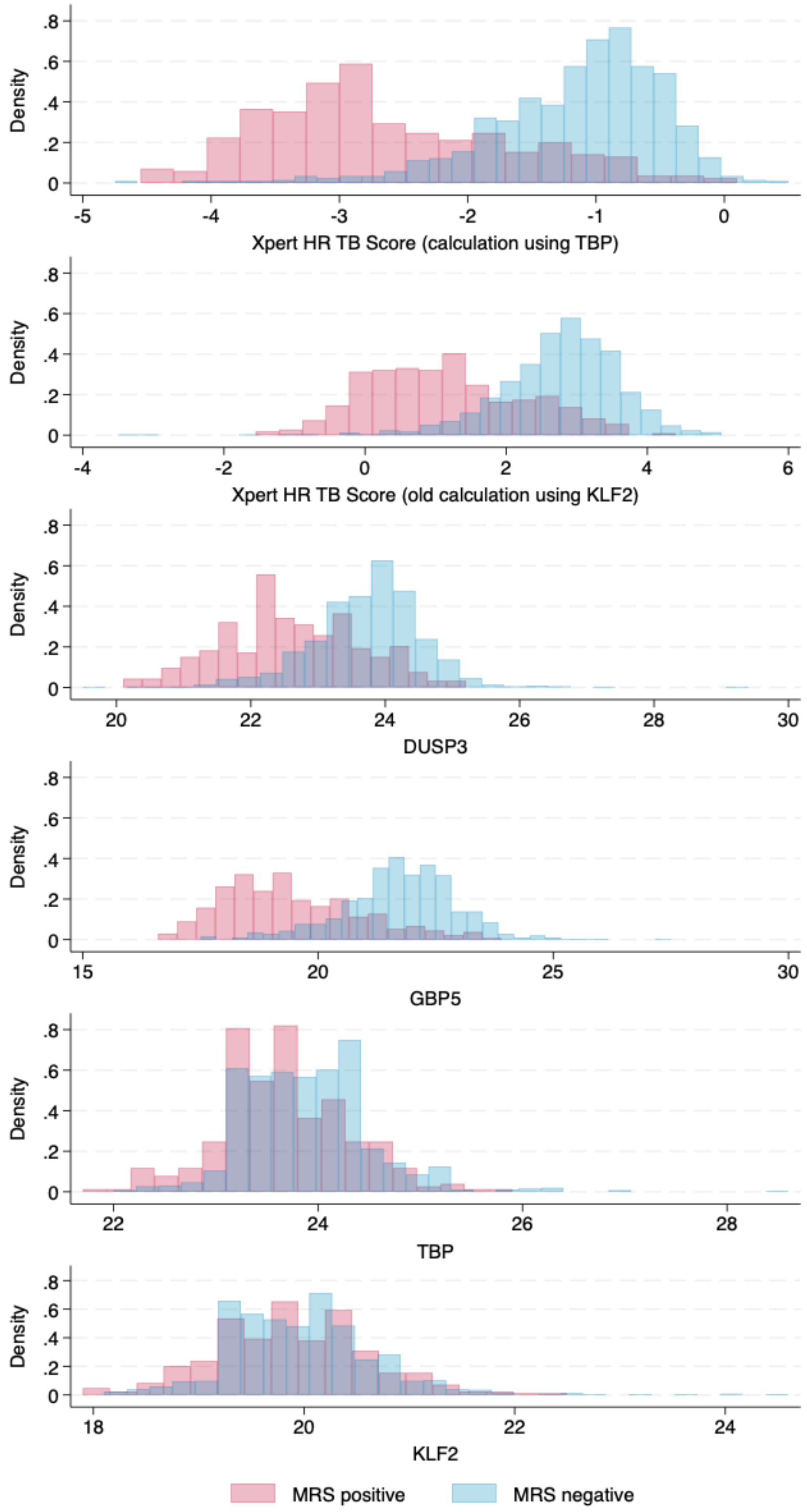

Xpert HR TB Score (panel 1) is calculated as (Ct *GBP5* + Ct *DUSP3*)/2 − Ct *TBP. A previous version of the assay used an alternate caluation using KLF2 in place of TBP (panel 2). Ct* Cycle threshold; *GBP5 guanylate binding protein 5; DUSP3 dual specificity phosphatase 3; KLF2 Krüppel-like factor 2; TBP TATA-binding protein; MRS microbiological reference standard. Median TB Score was -2.85 (IQR -3.28 to -2.18) for participants positive by MRS, and -1 (IQR -1.5 to -0.7) for participants negative by MRS.*

Figure S2. Xpert TB HR Receiver Operating Characteristic (ROC) curve using previous calculation against microbiologic reference standard.

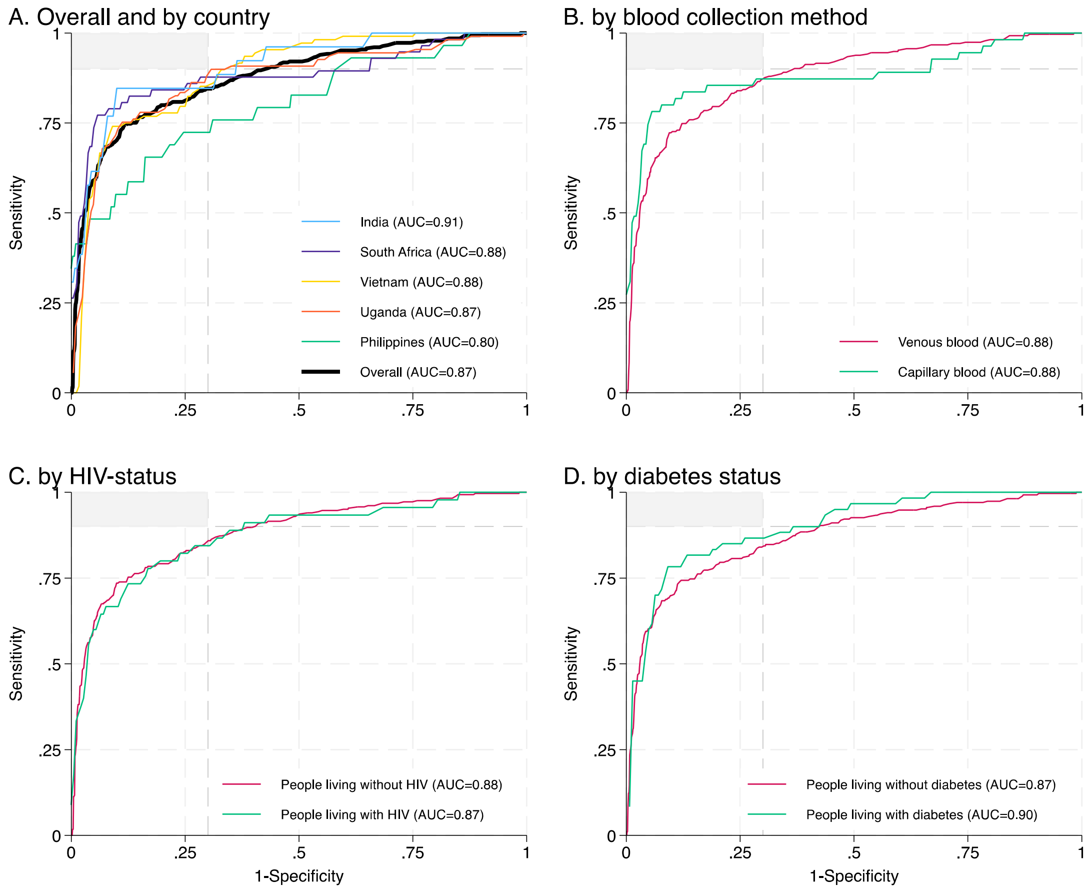

Previous studies calculated the TB score using KLF2 instead of TBP. Here we present the ROC curves using this calculation: Ct GBP5 + Ct DUSP3)/2 − Ct KLF2. The median TB Score calculated in this manner was 1.00 (IQR 0.30 to 1.9) for participants positive by MRS, and 2.85 (IQR 2.30 to -3.30) for participants negative by MRS. Using this calculation, the optimal cut-point defining a positive test in this population would be ≤2.7. Using this cut-point, sensitivity against the MRS is 90.3% (297/329, 95%CI 86.5-93.3), and specificity against the MRS is 56.8% (664/1170, 95% CI 53.9-59.6).

Table S4: Univariable regression between TB Score and other variables

		Coefficient	95% CI	R^2	p-value
**Country**	**Philippines**	0.71	0.56 - 0.86	0.072	<0.001
	**Vietnam**	0.48	0.33 -0 .63		
	**South Africa**	0.63	0.48 - 0.77		
	**Uganda**	0.63	0.48 - 0.77		
	**India**	0.25	0.10 - 0.40		
**Sex**	**Male**	0.13	0.03 - 0 .23	0.005	0.009
**Age, years**		-0.16	-0.31- 0.28	0.026	0.839
**BMI**		-0.05	-0.06 - -0.04	0.077	<0.001
**Person living with HIV[a]**	**Yes**	0.30	0.16 – 0.43	0.012	<0.001
**Diabetes**	**Yes**	0.13	-0.11 – 0.28	0.002	0.07
**Sputum Xpert semi-quantitative grade[b]**	**Trace**	-0.12	-0.73 – 0.49	0.097	<0.001
	**Low**	0.41	0.11 – 0.72		
	**Medium**	0.78	0.47 – 1.10		
	**High**	0.66	0.34 – 0.97		
**C-reactive protein**		0.01	0.01 – 0.01	0.24	<0.001

Linear regression to assess associations with TB Score, transformed to a positive value. N=1,499. Continuous variables assumed to be linear and based on a one unit increase. Categorical variables fitted as factors. [a]3 participants missing their HIV status. [b]303 participants with positive Xpert results included in this analysis

Table S5: Area Under the Receiver Operating Characteristic (ROC) curve, overall and by subgroups

		Number of observations	Area under the ROC curve	(95%CI)	χ^2 test p-value
**Overall**		1499	0.89	(0.86-0.91)	
**Country**	Philippines	325	0.83	(0.75-0.91)	0.244*
	Vietnam	284	0.88	(0.84-0.92)	0.938*
	South Africa	300	0.90	(0.84-0.95)	0.694*
	Uganda	314	0.87	(0.82-0.92)	0.463*
	India	276	0.91	(0.85-0.97)	0.448*
**Sex**	Male	813	0.87	(0.83-0.91)	0.375
	Female	686	0.89	(0.87-0.92)	
**Person living with HIV**	No	1267	0.88	(0.81-0.94)	0.774
	Yes	229	0.89	(0.87-0.91)	
**Diabetes**	No	1297	0.89	(0.84-0.95)	0.790
	Yes	202	0.88	(0.86-0.91)	

*Area under the ROC (AUROC) were compared using DeLong's test. *For country, the p-value is comparing the ROC for that country to the AUROC for the other countries.*

Table S6: Diagnostic accuracy of Xpert-HR by blood sampling method, stratified by HIV-status

	**Living with HIV**			**Not living with HIV**		
**Blood sampling method for Xpert-HR**	***N (row %)***	***Sensitivity (95% CI)***	***Specificity (95% CI)***	***N (row %)***	***Sensitivity (95% CI)***	***Specificity (95% CI)***
**Venous**	114 (9.4%)	95.8% (78.9 - 99.9%)	45.6% (35.0-56.4%)	1,099 (90.6%)	89.6% (85.1-93.1%)	66.1% (62.8-69.3%)
**Capillary**	115 (40.6)	95.2% (76.2 - 99.9%)	44.7% (34.4-55.3%)	168 (59.4%)	87.9% (71.8 - 96.6%)	64.4% (55.8-72.5%)

Capillary blood sampling occurred at only at the South Africa site. Using Chi-squared tests, the p-values for specificity comparing venous and capillary sampling is 0.891 for people living with HIV and 0.665 for people not living with HIV

Table S7: Cross tabulation of TB culture results and highest Xpert Ultra semi-quantitative grade for participants positive by the microbiological reference standard

	**TB culture not done**	**TB culture positive**	**TB culture negative**
**Trace**	3*	2	0
**Very low**	44	0	0
**Low**	95	0	0
**Medium**	76	0	0
**High**	79	0	0
**Xpert negative**	0	30	1,170

**3 patients had 2 Xpert Ultra trace results*

Table S8: Cross tabulation of TB culture results and highest Xpert Ultra semi-quantitative grade for participants positive by the microbiological reference standard

	**TB culture not done**	**TB culture positive**
**Trace**	3*	2
**Very low**	44	0
**Low**	95	0
**Medium**	76	0
**High**	79	0
**Xpert negative**	0	30

**3 patients had 2 Xpert Ultra trace results*

Figure S3: Positive and negative predictive values for the Xpert-HR and WHO minimum Target Product Profile accuracy for a range of TB prevalence

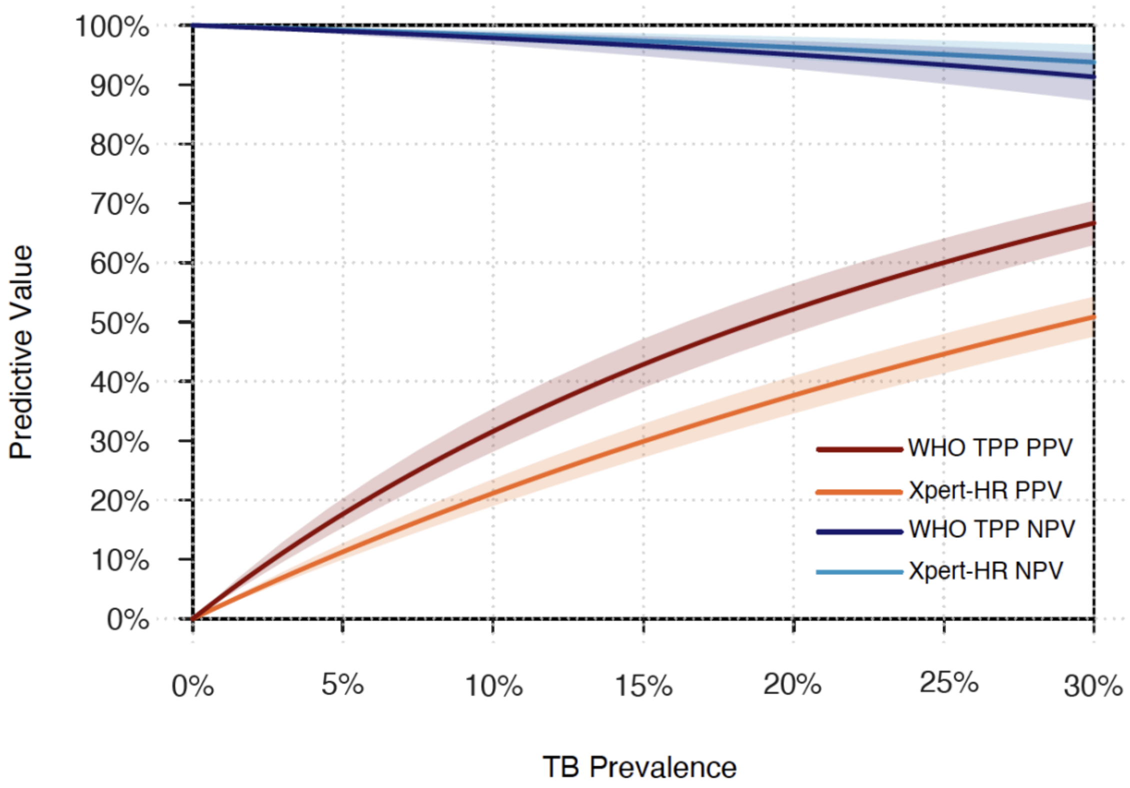

Positive and negative predictive values plotted for different TB prevalence, based on a cut-point of ≤-1.25 (i.e. sensitivity of 90.3% and specificity of 62.6%). The WHO TPP lines are based on a hypothetical triage test minimum criteria (sensitivity of 90% and specificity of 70%). Shaded areas represent 95% confidence intervals. Analysis done using 'plot-curve' in R. Xpert HR PPVs are 11.3%, 21.2%, 29.9% and 37.6%, and NPVs 99.2%, 98.3%, 97.3% and 96.3% for TB prevalence of 5%, 10%, 15% and 20% respectively. PPV positive predictive value; NPV negative predictive value; WHO World Health Organization; TPP Target Prodict Profile.

Figure S4: Numbers needing sputum testing based on a hypothetical cohort of 1000 patients with presumed TB and 10% TB prevalence

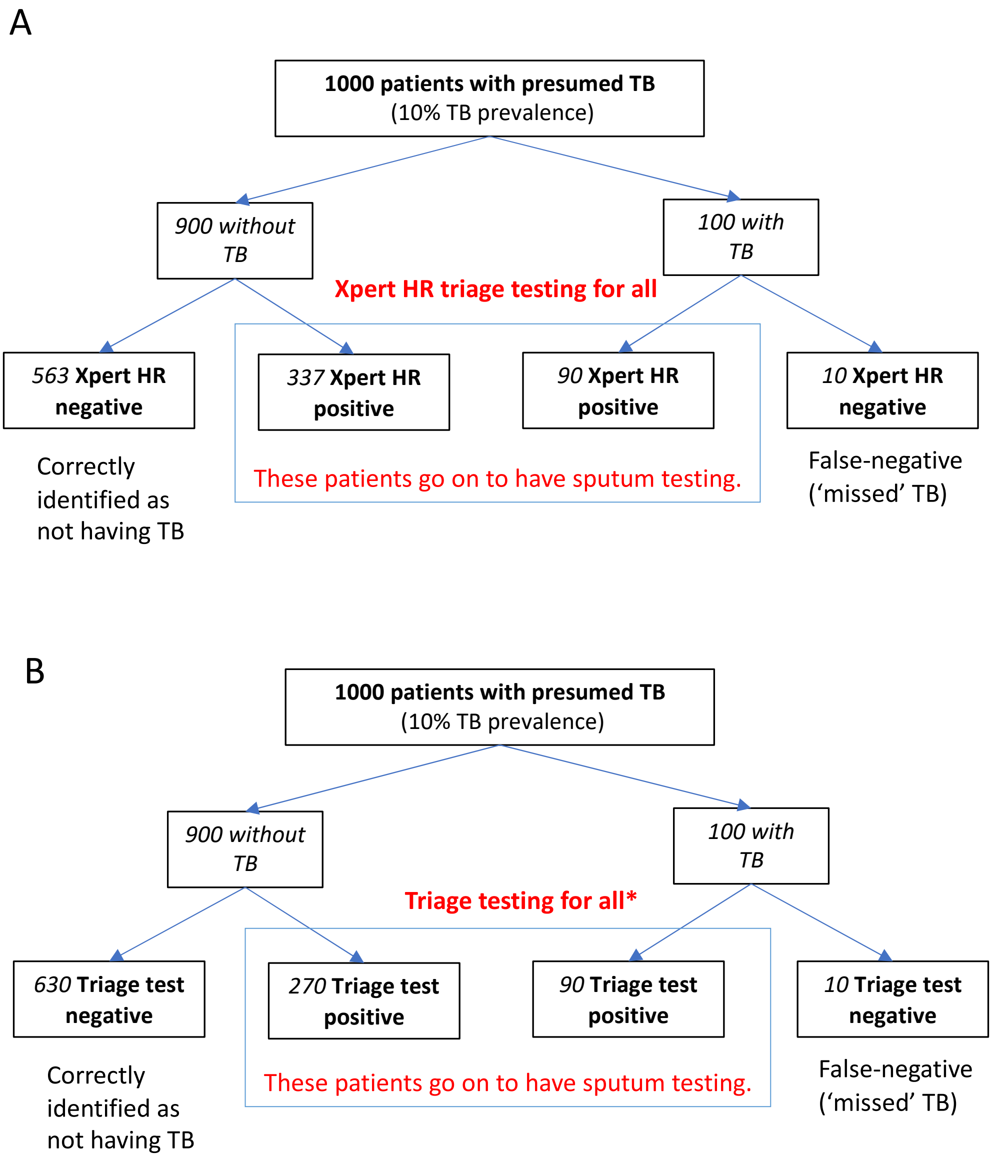

Panel A presents a hypothetical cohort tested with the Xpert-HR triage test. Panel B is based on a hypothetical triage test that meets WHO minimum TPP targets. Assumes all patients have valid test results.

Table S9: Diagnostic Accuracy for the Xpert-HR based on Microbiological Reference Standard overall and by subgroup

Category		TP	FP	FN	TN	TB prevalence (%)	Sensitivity (%)	(95%CI)	Specificity (%)	(95%CI)	PPV (%)	(95%CI)	NPV (%)	(95%CI)
**Overall (cut-point ≤-1.25)**		297	438	32	732	21.9	90.3	(86.5-93.3)	62.6	(59.7-65.3)	40.4	(36.8-44.1)	95.8	(94.1-97.1)
**Country**														
	**Philippines**	22	78	7	218	8.9	75.9	(56.5-89.7)	73.6	(68.2-78.6)	22.0	(14.3-31.4)	96.9	(93.6-98.7)
	**Vietnam**	102	82	6	94	38.0	94.4	(88.3-97.9)	53.4	(45.8-60.9)	55.4	(47.9-62.7)	94.0	(87.4-97.8)
	**South Africa**	52	105	5	138	19.0	91.2	(80.7-97.1)	56.8	(50.3-63.1)	33.1	(25.8-41.1)	96.5	(91.7-98.8)
	**Uganda**	97	75	12	130	34.7	89.0	(81.6-94.2)	63.4	(56.4-70.0)	56.4	(48.6-63.9)	91.5	(84.2-94.8)
	**India**	24	98	2	152	9.4	92.3	(74.9-99.1)	60.8	(54.4-66.9)	19.7	(13.0-27.8)	98.7	(95.2-99.8)
**Sex**														
	**Female**	203	210	20	380	15.5	88.7	(81.1-94.0)	60.7	(56.6-64.7)	29.2	(24.3-34.5)	96.7	(93.8-98.0)
	**Male**	94	228	12	352	27.4	91.0	(86.5-94.4)	64.4	(60.4-68.3)	49.2	(44.2-54.1)	95.0	(92.2-96.8)
**HIV status**														
	**HIV +ve**	43	101	2	83	19.7	95.6	(84.9-99.5)	45.1	(37.8-52.6)	29.9	(22.5-38.0)	97.6	(91.4-99.7)
	**HIV -ve**	253	336	30	648	22.3	89.4	(85.2-92.7)	65.9	(62.8-68.8)	43.0	(38.9-47.1)	95.6	(93.4-96.8)
**Diabetes**														
	**Yes**	53	44	7	98	29.7	88.3	(77.4-95.2)	69.0	(60.7-76.5)	54.6	(44.2-64.8)	93.3	(86.4-97.2)
	**No**	244	394	25	634	20.7	90.7	(86.6-93.9)	61.7	(58.6-64.7)	38.2	(34.5-42.1)	96.2	(94.1-97.3)
**Collection method**														
	**capillary**	50	101	5	130	19.2	90.9	(80.0-97.0)	56.3	(49.6-62.8)	33.1	(25.7-41.2)	96.3	(91.3-98.7)
	**venous**	247	337	27	602	22.6	90.1	(86.0-93.4)	64.1	(60.9-67.2)	42.3	(38.2-46.4)	95.7	(93.5-96.9)

TP True positive, FP False positive, FN False Negative, TN true negative, PPV positive predictive value, NPV negative predictive value

Figure S5: Decision Curve Analysis stratified by HIV status

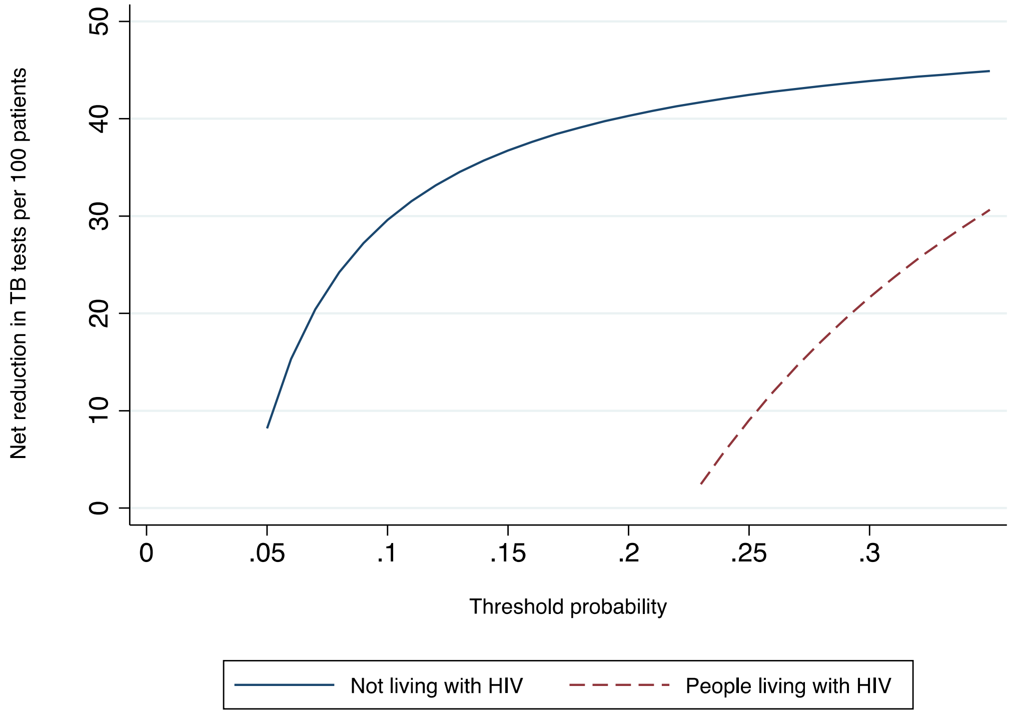

Decision curve analysis, shown as net reduction in (sputum-based) TB tests per 100 patients tested with Xpert-HR weighted by a range of threshold probabilities from 5 to 30%, stratified by HIV status. The threshold probability describes how clinicians and patients value different outcomes, and can be defined as the minimum probability of TB disease at which further testing would be warranted. A low threshold probability could reflect a clinician and/or patient who are very concerned about missing TB disease and therefore likely to do further TB testing. For example, a 5% threshold probability (equivalent to a risk:benefit odds of 1:19) where missing TB disease is 19 times worse than unnecessary testing, would lead to a net reduction of approximately 9 TB tests per 100 patients in people not living with HIV, but no net benefit in PLHIV. In contrast, clinicians and/or patients may be less worried about TB and not want to do further sputum-based testing, leading to a higher threshold probability. For example a 25% threshold probability, (equivalent to a risk:benefit odds of 1:3) the risk of missing TB is 3 times worse than unnecessary investigations for TB, and would lead to a net reduction in approximately 42 TB tests per 100 patients in people not living with HIV, but only 10 TB tests per 100 patients in PLHIV.

Table S8: Demographical and clinical characteristics overall and by country for secondary analysis using Sputum Xpert Reference Standard

		Overall	Philippines	Vietnam	South Africa	Uganda	India
**N**		1606	352	303	340	328	283
**Sex**	**Male**	874 (54.4%)	156 (44.3%)	187 (61.7%)	173 (50.9%)	189 (57.6%)	169 (59.7%)
	**Female**	732 (45.6%)	196 (55.7%)	116 (38.3%)	167 (49.1%)	139 (42.4%)	114 (40.3%)
**Age, mean (SD), years**		42.5 (15.5)	40.1 (15.4)	51.6 (15.9)	40.0 (12.2)	34.9 (12.7)	47.5 (15.5)
**BMI, median (IQR)**		21.5 (19.1, 25.2)	23.6 (20.2, 27.5)	20.2 (18.7, 21.9)	22.3 (19.4, 28.3)	20.8 (19.0, 24.0)	22.4 (19.0, 26.0)
**Cough for 2 weeks**		1606 (100.0%)	352 (100.0%)	303 (100.0%)	340 (100.0%)	328 (100.0%)	283 (100.0%)
**Fever**		568 (35.4%)	51 (14.5%)	106 (35.0%)	123 (36.2%)	222 (67.7%)	66 (23.3%)
**Weight loss**		754 (46.9%)	99 (28.1%)	88 (29.0%)	218 (64.1%)	226 (68.9%)	123 (43.5%)
**Night Sweats**		566 (35.2%)	57 (16.2%)	94 (31.0%)	188 (55.3%)	201 (61.3%)	26 (9.2%)
**Prior TB diagnosis[b]**	**Yes**	316 (19.7%)	46 (13.1%)	63 (20.8%)	127 (37.4%)	41 (12.5%)	39 (13.8%)
**Person living with HIV[a]**	**Yes**	254 (15.8%)	3 (0.9%)	2 (0.7%)	138 (40.8%)	101 (30.8%)	10 (3.5%)
	**Median CD4, cells/µL (IQR)**	396.0 (204.0, 654.0)	527.0 (184.0, 812.0)	547.0 (497.0, 597.0)	402.5 (205.0, 667.0)	355.0 (200.0, 613.0)	587.0 (220.0, 692.0)
**Diabetes**	**Yes**	213 (13.3%)	46 (13.1%)	71 (23.4%)	19 (5.6%)	26 (7.9%)	51 (18.0%)
**Sputum Xpert status[c]**	**Negative**	1309 (81.5%)	330 (93.8%)	207 (68.3%)	291 (85.6%)	222 (67.7%)	259 (91.5%)
	**Positive**	297 (18.5%)	22 (6.2%)	96 (31.7%)	49 (14.4%)	106 (32.3%)	24 (8.5%)
**TB Microbiologic reference standard**	**TB Negative**	1165 (72.8%)	296 (84.1%)	176 (58.1%)	241 (70.9%)	203 (61.9%)	249 (89.6%)
	**TB Positive**	327 (20.4%)	29 (8.2%)	108 (35.6%)	56 (16.5%)	109 (33.2%)	25 (9.0%)
	**Indeterminate**	109 (6.8%)	27 (7.7%)	19 (6.3%)	43 (12.6%)	16 (4.9%)	4 (1.4%)
**Venous or capillary blood collection**	**Capillary**	325 (20.2%)	0 (0.0%)	0 (0.0%)	325 (95.6%)	0 (0.0%)	0 (0.0%)
	**Venous**	1281 (79.8%)	352 (100.0%)	303 (100.0%)	15 (4.4%)	328 (100.0%)	283 (100.0%)

[a]3 participants were unsure about prior TB diagnoses. [b]3 participants had an unknown HIV status. [c]1 participant was missing a sputum Xpert result.

Table S9: Diagnostic Accuracy for the Xpert-HR based on Sputum Xpert Reference Standard overall and by subgroup

Category		TP	FP	FN	TN	TB prevalence (%)	Sensitivity (%)	(95%CI)	Specificity (%)	(95%CI)	PPV (%)	(95%CI)	NPV (%)	(95%CI)
**Overall (cut-point <-1.45)**		908	29	401	268	18.5	90.2	(86.3-93.4)	69.4	(66.8-71.9)	40.1	(36.3-43.9)	96.9	(95.6-97.9)
**Country**														
	**Philippines**	267	4	63	18	6.2	81.8	(59.7-94.8)	80.9	(76.2-85.0)	22.2	(13.7-32.8)	98.5	(96.3-99.6)
	**Vietnam**	132	7	75	89	31.7	92.7	(85.6-97.0)	63.8	(56.8-70.3)	54.3	(46.3-62.1)	95.0	(89.9-98.0)
	**South Africa**	180	5	111	44	14.4	89.8	(77.8-96.6)	61.9	(56.0-67.5)	28.4	(21.4-36.2)	97.3	(93.8-99.1)
	**Uganda**	152	10	70	96	32.3	90.6	(83.3-95.4)	68.5	(61.9-74.5)	57.8	(49.9-65.4)	93.8	(88.9-97.0)
	**India**	177	3	82	21	8.5	87.5	(67.6-97.3)	68.3	(62.3-74.0)	20.4	(13.1-29.5)	98.3	(95.2-99.7)
**Sex**														
	**Female**	473	21	197	183	12.7	91.4	(83.8-96.2)	68.1	(64.3-71.7)	29.4	(24.2-35.0)	98.2	(96.5-99.2)
	**Male**	435	8	204	85	23.3	89.7	(84.7-93.5)	70.6	(67.0-74.0)	48.2	(43.0-53.3)	95.7	(93.6-97.3)
**HIV status**														
	**HIV +ve**	801	27	290	232	15.0	94.7	(82.3-99.4)	49.1	(42.2-55.9)	24.7	(17.9-32.5)	98.1	(93.5-99.8)
	**HIV -ve**	106	2	110	36	19.2	89.6	(85.2-93.0)	73.4	(70.7-76.0)	44.4	(40.1-48.8)	96.7	(95.3-97.8)
**Diabetes**														
	**Yes**	795	23	356	219	25.8	89.1	(77.8-95.9)	71.5	(63.8-78.4)	52.1	(41.6-62.5)	95.0	(89.3-98.1)
	**No**	113	6	45	49	17.4	90.5	(86.1-93.9)	69.1	(66.3-71.7)	38.1	(34.1-42.2)	97.2	(95.8-98.2)
**Collection method**														
	**capillary**	172	5	105	43	14.8	89.6	(77.3-96.5)	62.1	(56.1-67.8)	29.1	(21.9-37.1)	97.2	(93.5-99.1)
	**venous**	172	5	105	43	19.4	90.4	(86.0-93.7)	71.3	(68.5-74.1)	43.2	(38.9-47.6)	96.8	(95.3-98.0)

TP True positive, FP False positive, FN False Negative, TN true negative, PPV positive predictive value, NPV negative predictive value, CI confidence interval

Figure S5. Xpert TB HR Receiver Operating Characteristic (ROC) curve analysis against sputum Xpert reference standard.

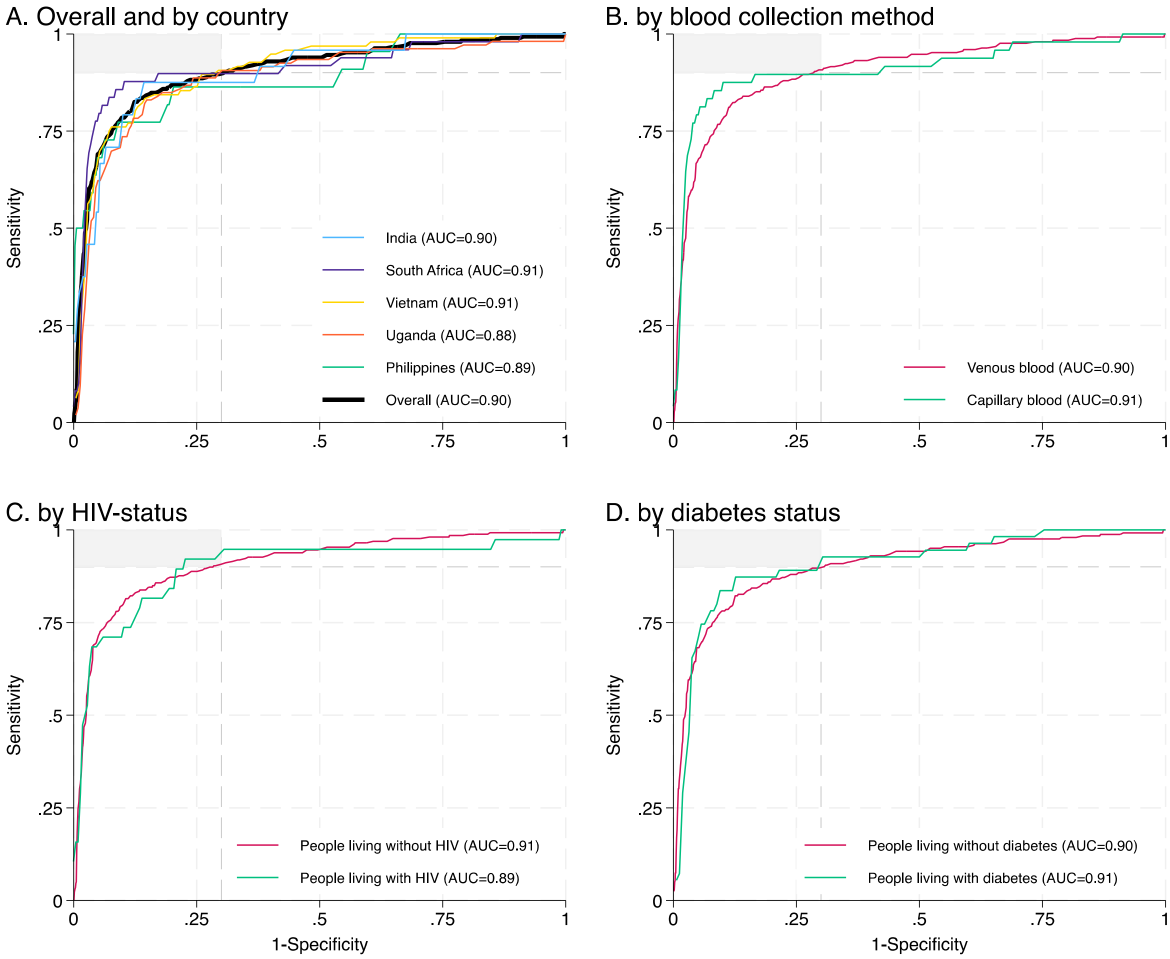

N=1606 participants with valid sputum Xpert Ultra results. Median TB Score was -2.90 (IQR -3.35 to -2.20) for participants positive by SXRS, and -1.05 (IQR -1.55 to -0.70) for participants negative by SXRS.

STARD checklist

Section & Topic	No	Item	Reported on page #
**TITLE OR ABSTRACT**	**1**	Identification as a study of diagnostic accuracy using at least one measure of accuracy (such as sensitivity, specificity, predictive values, or AUC)	1&2
**ABSTRACT**	**2**	Structured summary of study design, methods, results, and conclusions (for specific guidance, see STARD for Abstracts)	3
**INTRODUCTION**			
	**3**	Scientific and clinical background, including the intended use and clinical role of the index test	5
	**4**	Study objectives and hypotheses	5
**METHODS**			
Study design	**5**	Whether data collection was planned before the index test and reference standard were performed (prospective study) or after (retrospective study)	6
Participants	**6**	Eligibility criteria	6
	**7**	On what basis potentially eligible participants were identified (such as symptoms, results from previous tests, inclusion in registry)	6
	**8**	Where and when potentially eligible participants were identified (setting, location and dates)	6
	**9**	Whether participants formed a consecutive, random or convenience series	6
Test methods	**10a**	Index test, in sufficient detail to allow replication	6
	**10b**	Reference standard, in sufficient detail to allow replication	6-7
	**11**	Rationale for choosing the reference standard (if alternatives exist)	NA
	**12a**	Definition of and rationale for test positivity cut-offs or result categories of the index test, distinguishing pre-specified from exploratory	7
	**12b**	Definition of and rationale for test positivity cut-offs or result categories of the reference standard, distinguishing pre-specified from exploratory	7
	**13a**	Whether clinical information and reference standard results were available to the performers/readers of the index test	7

	**13b**	Whether clinical information and index test results were available to the assessors of the reference standard	7
Analysis	**14**	Methods for estimating or comparing measures of diagnostic accuracy	7
	**15**	How indeterminate index test or reference standard results were handled	7
	**16**	How missing data on the index test and reference standard were handled	7
	**17**	Any analyses of variability in diagnostic accuracy, distinguishing pre-specified from exploratory	7
	**18**	Intended sample size and how it was determined	7
**RESULTS**			
Participants	**19**	Flow of participants, using a diagram	8, Figure 1
	**20**	Baseline demographic and clinical characteristics of participants	9
	**21a**	Distribution of severity of disease in those with the target condition	9/Appendix
	**21b**	Distribution of alternative diagnoses in those without the target condition	NA
	**22**	Time interval and any clinical interventions between index test and reference standard	12
Test results	**23**	Cross tabulation of the index test results (or their distribution) by the results of the reference standard	Appendix, table S7
	**24**	Estimates of diagnostic accuracy and their precision (such as 95% confidence intervals)	10/ Figure 3/ Appendix table S9
	**25**	Any adverse events from performing the index test or the reference standard	NA
**DISCUSSION**			
	**26**	Study limitations, including sources of potential bias, statistical uncertainty, and generalisability	16-17
	**27**	Implications for practice, including the intended use and clinical role of the index test	16-17
**OTHER INFORMATION**			
	**28**	Registration number and name of registry	ClinicalTrials.gov Identifier: NCT04923958
	**29**	Where the full study protocol can be accessed	On request from authors
	**30**	Sources of funding and other support; role of funders	8, 18
